# Assessing the role of live poultry trade in community-structured transmission of avian influenza in China

**DOI:** 10.1073/pnas.1906954117

**Published:** 2020-03-02

**Authors:** Qiqi Yang, Xiang Zhao, Philippe Lemey, Marc A. Suchard, Yuhai Bi, Weifeng Shi, Di Liu, Wenbao Qi, Guogang Zhang, Nils Chr. Stenseth, Oliver G. Pybus, Huaiyu Tian

**Affiliations:** ^a^State Key Laboratory of Remote Sensing Science, College of Global Change and Earth System Science, Beijing Normal University, 100875 Beijing, China;; ^b^National Institute for Viral Disease Control and Prevention, Collaboration Innovation Center for Diagnosis and Treatment of Infectious Diseases, Chinese Center for Disease Control and Prevention, Key Laboratory for Medical Virology, National Health and Family Planning Commission, 102206 Beijing, China;; ^c^Department of Microbiology, Immunology and Transplantation, Rega Institute, Clinical and Epidemiological Virology, KU Leuven, 3000 Leuven, Belgium;; ^d^Department of Biomathematics, David Geffen School of Medicine, University of California, Los Angeles, CA 90095;; ^e^Department of Human Genetics, David Geffen School of Medicine, University of California, Los Angeles, CA 90095;; ^f^Department of Biostatistics, UCLA Fielding School of Public Health, University of California, Los Angeles, CA 90095;; ^g^Shenzhen Key Laboratory of Pathogen and Immunity, Guangdong Key Laboratory for Diagnosis and Treatment of Emerging Infectious Diseases, State Key Discipline of Infectious Disease, Second Hospital Affiliated to Southern University of Science and Technology, Shenzhen Third People’s Hospital, 518112 Shenzhen, China;; ^h^Key Laboratory of Pathogenic Microbiology and Immunology, Institute of Microbiology, Center for Influenza Research and Early-Warning (CASCIRE), Chinese Academy of Sciences, 10010 Beijing, China;; ^i^Institute of Pathogen Biology, Taishan Medical College, 271000 Taian, Shandong, China;; ^j^Key Laboratory of Special Pathogens and Biosafety, Wuhan Institute of Virology, Chinese Academy of Sciences, 430071 Wuhan, Hubei, China;; ^k^National and Regional Joint Engineering Laboratory for Medicament of Zoonoses Prevention and Control, College of Veterinary Medicine, South China Agricultural University, 510642 Guangzhou, China;; ^l^Research Institute of Forest Ecology, Environment and Protection, National Bird Banding Center of China, Chinese Academy of Forestry, Key Laboratory of Forest Protection of State Forestry and Grassland Administration, 100091 Beijing, China;; ^m^Centre for Ecological and Evolutionary Synthesis (CEES), Department of Biosciences, University of Oslo, N-0316 Oslo, Norway;; ^n^Department of Zoology, University of Oxford, OX1 3PS Oxford, United Kingdom;; ^o^Department of Pathobiology and Population Sciences, The Royal Veterinary College, AL9 7TA London, United Kingdom

**Keywords:** avian influenza, poultry trade, phylogeography, community-structured transmission

## Abstract

The live poultry trade is thought to play an important role in the spread and maintenance of highly pathogenic avian influenza A viruses (HP AIVs) in Asia. Despite an abundance of small-scale observational studies, the role of the poultry trade in disseminating AIV over large geographic areas is still unclear, especially for developing countries with complex poultry production systems. Here we combine virus genomes and reconstructed poultry transportation data to measure and compare the spatial spread in China of three key subtypes of AIV: H5N1, H7N9, and H5N6. Although it is difficult to disentangle the contribution of confounding factors, such as bird migration and spatial distance, we find evidence that the dissemination of these subtypes among domestic poultry is geographically continuous and likely associated with the intensity of the live poultry trade in China. Using two independent data sources and network analysis methods, we report a regional-scale community structure in China that might explain the spread of AIV subtypes in the country. The identification of this structure has the potential to inform more targeted strategies for the prevention and control of AIV in China.

## Significance

The emergence and transmission of avian influenza viruses (AIVs) pose a threat to public health and result in enormous economic losses. Here we discover an association between the community structure of the poultry trade network and AIV transmission in China by combining virus genomes and statistical modeling of the poultry trade. Importantly, we are able to “replicate” this finding by comparing the dynamics of three strains of AIV (H5N1, H7N9, and H5N6) that currently cocirculate in poultry in China. Given the detection of a continuous process of AIV geographic spread among poultry, our results indicate that at the national scale there are repeatable and potentially predictable patterns that can be used to shape future strategies for AIV control and prevention.

H5N1 highly pathogenic avian influenza A virus (HP AIV) was first identified in 1996 (1). In the following two decades, it has circulated among various bird species and spread to more than 60 countries (2). Domestic poultry are thought to play an important role in the transmission and spread of H5N1 HP AIV and the virus’s ability to occasionally infect humans means it poses a significant public health risk (3, 4). Human infection with H5N1 HP AIV results in an estimated fatality rate of 50 to 60% (5), and most cases are linked directly or indirectly to exposure to live poultry (4). Maintenance of the virus in domestic poultry hosts has enabled further virus evolution and the emergence of novel influenza viruses of avian origin. H5N1 HP AIV emerged in part from the adaptation of low-pathogenicity AIVs from waterfowl to domestic poultry hosts (6). More recently, two novel reassortant H5 AIV subtypes, H5N6 and H5N8, have emerged in Asia (7, 8); the latter subtype subsequently spread to Europe, North America, and Africa, causing outbreaks in local poultry (9). However, the mechanisms by which these viruses disseminate and cause repeated large-scale waves of infection in domestic poultry remain unclear.

Previous studies have explored the association between AIV transmission and the trade in live poultry and associated poultry products, but empirical data on transmission dynamics are scarce. Although many retrospective epidemiological studies have assessed common risk factors for AIV disease outbreaks, comparatively few have include poultry trade patterns, which are considered difficult to obtain (10). Some studies (11–13) have attempted to infer poultry trade networks at the local level by summarizing the routes of traders among live poultry markets (LPMs), and have concluded that poultry trade patterns are associated with AIV outbreaks. Other studies have investigated AIV genome sequences using Bayesian phylogeographic methods (14, 15) and have found that poultry population density and the number of poultry markets are determinants of the spatial diffusion of AIVs in endemic areas (16). However, it can be difficult to infer underlying transmission processes from such data due to the uneven sampling of virus genomes through time and space, and a reliance on passive surveillance means that many virus lineages may go unsampled. The paucity of data on the poultry trade and variable virus genome sampling therefore combine to hinder our understanding of the factors that facilitate the dissemination of H5N1 and other AIV subtypes over large geographic scales, especially in countries with high volumes of poultry production and trade sectors that are not strongly regulated (17).

Here we combine poultry trade movements (inferred using a gravity model) in China with large-scale analysis of AIV genome sequences in order to reconstruct hidden virus transmission routes and test the hypothesis that the live poultry trade in China affects the spread of HP AIV in the country. We reconstruct the estimated transmission dynamics in China of HPAI H5N1 from 1996, and compare them with the dynamics of other, more recently emerged, HP AIV lineages. We find that AIV transmission patterns can be explained by a national-level community structure of the poultry trade network in China. Our results provide insights into the large-scale structure and repeatability of AIV lineage emergence in Asia and may be useful in predicting the emergence of other AIV strains in other regions.

## Results

### The Spread of H5N1, H7N9, and H5N6 in China.

**Fig. 1.**
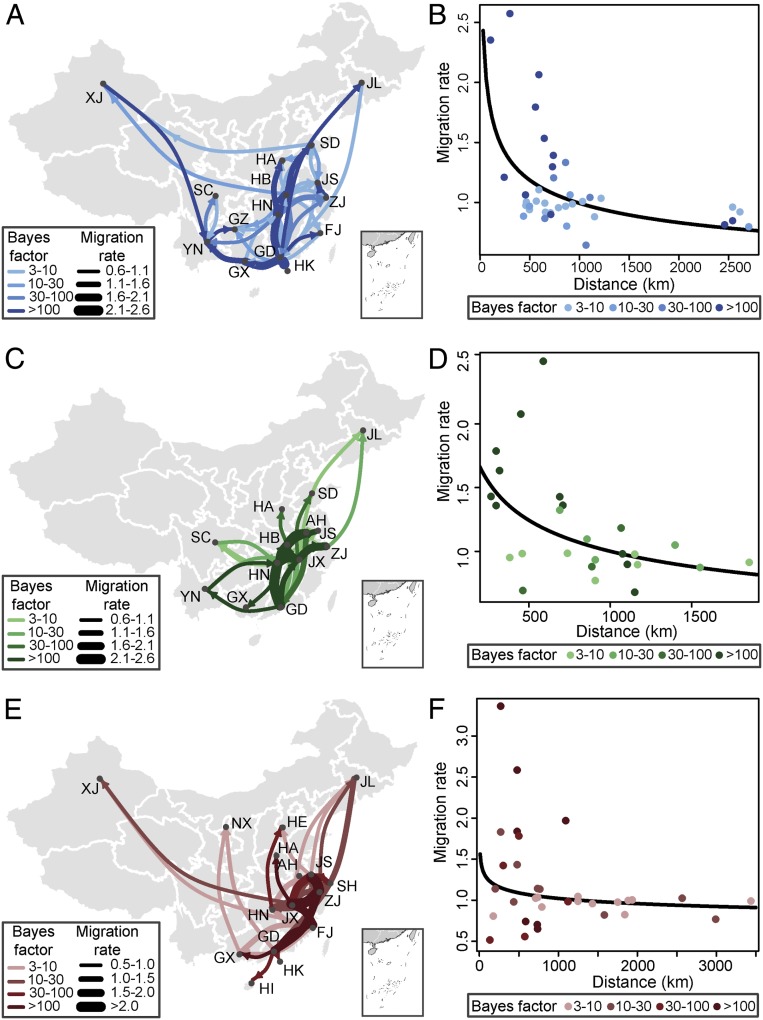
Spatiotemporal dissemination of AIV subtypes H5N1, H5N6, and H7N9 among poultry in China, determined by Bayesian phylogeography inference of HA gene sequences. (*A*, *C*, and *E*) Curves show the among-province virus lineage transitions statistically supported with Bayes factor >3 for subtypes H5N1 (*A*), H5N6 (*C*), and H7N9 (*E*). Curve widths represent transition rate values; curve colors represent corresponding statistical support (Bayes factor value) for each transition rate (*Inset*). (*B*, *D*, and *F*) Among-province virus lineage transition rates (supported with Bayes factor >3) decrease with geodesic distance between provinces for subtypes H5N1 (*B*), H5N6 (*D*), and H7N9 (*F*).

The hemagglutinin (HA) gene phylogenies of AIV subtypes H5N1, H7N9, and H5N6 in China exhibit frequent virus lineage movements among locations, indicating substantial geographic mixing (*SI Appendix*, Fig. S1). Taking H5N1 as an example, a discrete phylogeographic analysis of HPAI H5N1 in Chinese poultry revealed many well-supported viral lineage movements among provinces (as previously noted in ref. 16). In addition to this general pattern, we further observed that lineage movements occur between pairs of provinces that are adjacent or close (Fig. 1*A*). Consequently, lineage transition rates between locations are inversely related to distance, suggesting a more geographically continuous process of virus spread in poultry in China (Fig. 1*B*). This pattern is consistent for all three AIV subtypes (Fig. 1). However, some long-distance movement events were also observed, notably to or from the northern provinces of Jilin, Xinjiang (H5N1 and H7N9), and Ningxia (H7N9 only). Since virus sequences are unavailable for many of the provinces that directly neighbor Jilin, Xinjiang, and Ningxia, it is possible that AIV is present but underreported in these intermediate locations, and that virus movements occur over shorter distances.

**Fig. 2.**
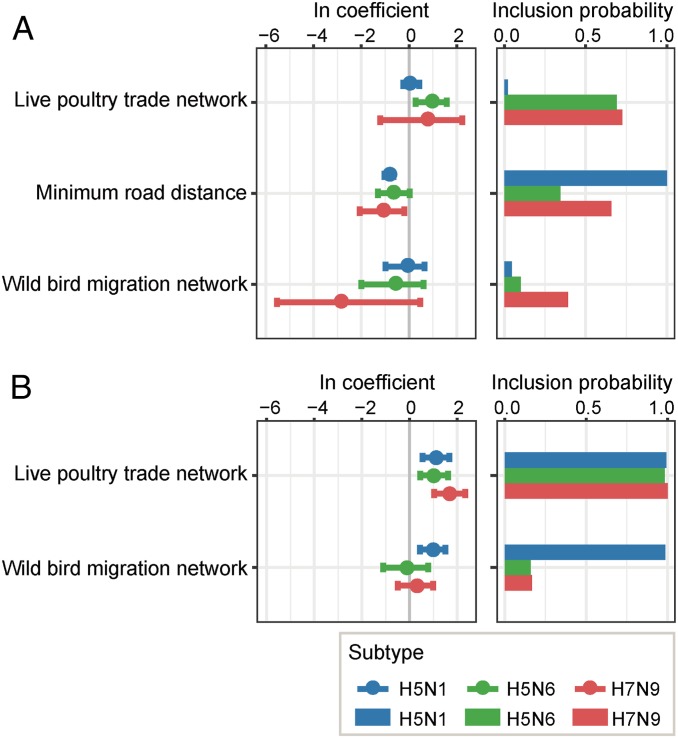
Contributions of predictor variables to the dissemination of H5N1, H5N6, and H7N9 lineages in poultry in China, determined from analysis of HA gene sequences. (*A*) Results when the minimum road network distance predictor is included in the analysis. (*B*) Results when the minimum road network distance predictor is excluded from the analysis. AIV subtypes H5N1, H5N6, and H7N9 are shown as blue, green and red, respectively. (*Left*) Circles show the estimated conditional effect sizes for the predictor coefficients (>0 = positive association, <0 = negative association). Error bars represent the 95% highest posterior density (HPD) credible interval for these estimates. (*Right*) Bars show the posterior probability of inclusion of each predictor in the model.

Since all three AIV subtypes in Chinese poultry exhibited evidence of a continuous process of geographic spread, we sought to test which variables might be associated with AIV lineage movements in China, with the aim of informing prevention and control of the disease. To do so, we employed a Bayesian phylogeographic approach that uses a generalized linear model (GLM) to quantify the contribution of predictor variables to the among-province lineage transition rates. This method was applied to HA sequences from the three independent AIV lineages in China: H5N1, H7N9, and H5N6. To begin, we used the established procedure of considering all potential predictors concurrently. This analysis revealed that many potential predictors are not associated with viral lineage movement (*SI Appendix*, Figs. S2 and S3). Based on the results of fully specified models, with and without distance, and sensitivity analysis (*SI Appendix*, Fig. S4), we recomputed these phylogeographic analyses using a much smaller set of predictors, specifically those that have been hypothesized by others to affect the large-scale dissemination of AIV (6, 8, 16, 18): 1) the intensity of the poultry trade among locations in China, 2) the migration of wild birds among locations in China, and 3) distances along the road network in China (see *Methods* for details). Although minimum road distance is strongly inversely associated with the dissemination of AIV lineages across a range of model configurations (Fig. 2*A*), it is likely that this predictor is simply acting as a proxy for the spatial distance component of the other two predictors. Since our primary hypothesis relates to the linkage of the poultry trade network and the wild bird migration network to AIV dissemination in China, we excluded the minimum road distance predictor from the previous exploratory analyses (Fig. 2*B*). We found that for all three subtypes (H5N1, H7N9, and H5N6), the live poultry trade network is positively associated with viral lineage spread, although this association is considerably less certain for H5N1 than for the other two subtypes (Fig. 2*B*). In contrast, the wild bird migration network is associated only with dissemination of H5N1 in China and not with the other two subtypes (Fig. 2*B*). The robustness of this result was confirmed using a model that directly compares the model inclusion probability of the wild bird migration and poultry trade predictors (*SI Appendix*, Table S1). Following these results, subsequent network analyses of these independent datasets were undertaken to explore how the AIV diffusion process is shaped by the structure of the live poultry trade network.

### Effect of LPTC Structure on the Spread of AIV in Poultry in China.

**Fig. 3.**
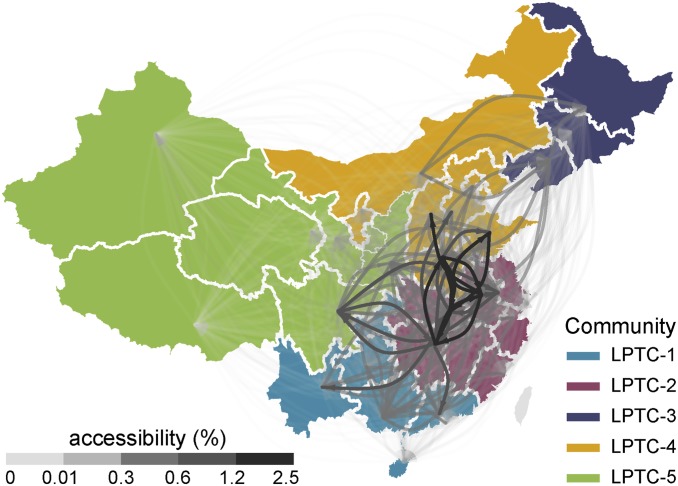
Estimated poultry trade among provinces and inferred subnational communities in the poultry trade network in China. The gray/black curves represent the accessibility of live poultry trade flows between pairs of provinces. Light gray and dark gray curves represent low and high accessibility flows, respectively. Provinces are colored according to the subnational live poultry trade community structure, identified using the Walktrap community-finding algorithm. LPTC = live-poultry trade community. The five communities are shown in blue, magenta, purple, mustard, and green, respectively.

The analysis above found that the poultry trade network in China can predict the risk of AIV lineage spread from one location to another, especially for the H7N9 and H5N6 subtypes. Thus, the epidemiological consequences of viral introduction and spread should depend on the structure of the poultry trade network at the national scale. To understand and quantify this structure, and to identify subnational poultry trade communities, we used a Walktrap algorithm to estimate a maximal-modularity subdivision of the poultry trade network in China (Fig. 3). This procedure identified five live poultry trade communities (LPTCs), that correspond spatially to the south (LPTC-1), east (LPTC-2), northeast (LPTC-3), midnorth (LPTC-4), and west (LPTC-5) of China (Fig. 3). The LPTC community structure is robust to perturbation (Dataset S1). This result indicates that transmission of AIV lineages in China may be community-structured, such that virus dissemination between localities within a community is more likely than dissemination among communities.

**Fig. 4.**
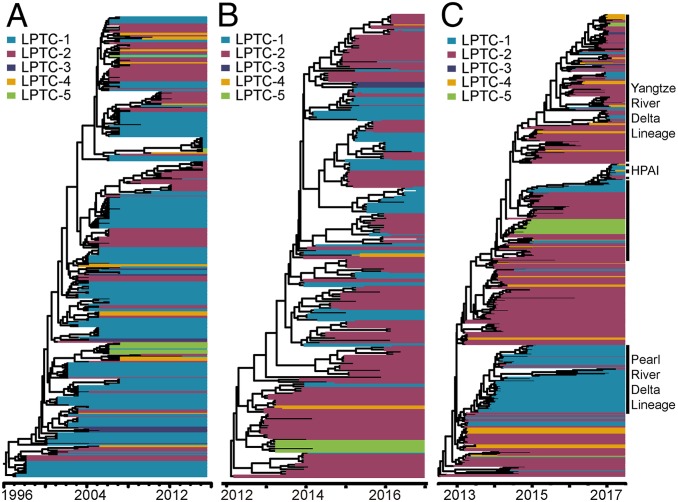
Spatial dynamics of H5N1, H5N6, and H7N9 AIVs. LPTCs 1 through 5 are shown in blue, magenta, purple, mustard, and green, respectively. (*A*) Maximum clade credibility trees of the H5N1 virus isolated from poultry in China. (*B*) Maximum clade credibility trees of H5N6 virus isolated from poultry in China. (*C*) Maximum clade credibility trees of H7N9 virus. To illustrate a wider geographical distribution of H7N9, we used 444 HA sequences of H7N9 viruses isolated from poultry, the environment, and humans in China.

In order to assess whether the transmission of AIV lineages belonging to all three subtypes are indeed associated with poultry trade communities in China, we undertook new discrete Bayesian phylogenetic analyses of the HA sequences belonging to AIV subtypes H5N1, H7N9, and H5N6. In these phylogeographic analyses, sequence locations were encoded using the five LPTCs. The LPTCs recapitulate important events in the phylogenetic history of all three subtypes (Fig. 4). The H5N1 subtype initially became enzootic in southern China (LPTC-1) (19), and major clades subsequently spread to southeastern China (LPTC-2) and midnorthern China (LPTC-4) (20) (Fig. 4*A*). A similar pattern of community-structured circulation was observed for H7N9 and H5N6 (Fig. 4 *B* and *C*). Originating from southeastern China (LPTC-2), H7N9 viruses diverged into two monophyletic groups, the Yangtze River Delta lineage and the Pearl River Delta lineage (21), which coincide with LPTC-2 and LPTC-1 in our LPTC network. H7N9 AIV has circulated in these regions for more than three years, causing epidemic waves I through IV (22). However, in epidemic wave V, H7N9 was found to have spread from LPTC-1 to all other LPTCs in China (22). For H5N6, most monophyletic groups of viruses have circulated regionally within LPTC-1 and LPTC-2 (7), although AIV lineage movements among LPTCs are increasing (Fig. 4*C*).

**Fig. 5.**
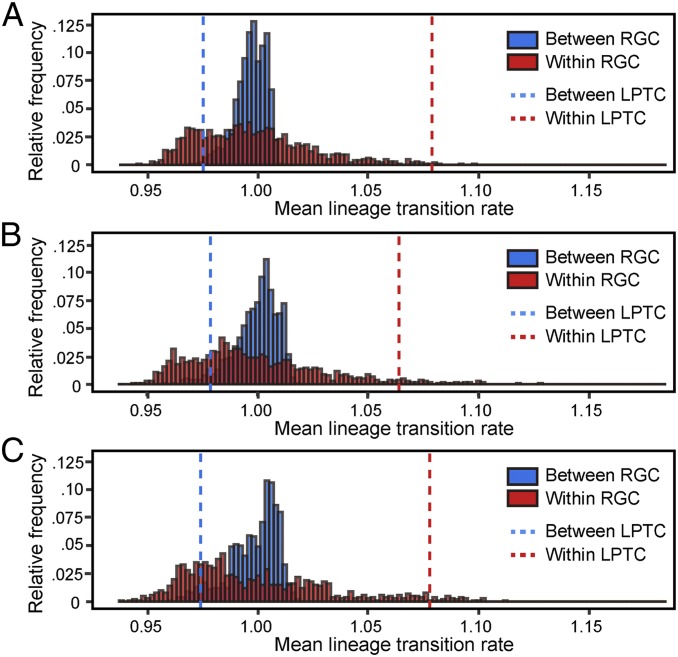
Histograms of mean among-location AIV lineage transition rates. The raw transition rates between locations were obtained from the analyses reported in Fig. 1. Shown are two means: blue = mean transition rate for pairs of locations in LPTCs; red = mean transition rate for pairs of locations in the same LPTC. Dotted vertical lines show the two means calculated using the empirically derived (true) LPTC network (Fig. 3). The histograms show the same mean values, calculated using 1,000 RGCs (see *Methods* for details). Results shown for (*A*) H5N1, (*B*) H5N6, and (*C*) H7N9.

We performed two subsequent analyses to explore whether the LPTCs explain the dissemination of AIV in China. First, we examined the estimated among-province lineage transition rates (i.e., those reported in Fig. 1 for all three AIV subtypes). We found that transition rates between provinces in the same LPTC (H5N1 mean = 1.08; H5N6 mean = 1.06; H7N9 mean = 1.07) were on average higher than those for provinces in different LPTCs (H5N1 mean = 0.98; H5N6 mean = 0.98; H7N9 mean = 0.97). Second, we conducted a randomization test in which locations (i.e., provinces) were randomly assigned to five communities (with the same number of members as our empirical LPTCs). For each randomly generated community (RGC), we calculated the mean within- and among-community lineage transition rates between locations (as above). This randomization procedure was repeated 1,000 times. As expected, in the randomly generated communities there was no difference between the mean transition rate for location pairs in the same community versus the mean transition rate for location pairs in different communities (Fig. 5). The results in Fig. 5 demonstrate that the LPTC in Fig. 3 does indeed contribute to the pattern of AIV lineage movement in China.

The subnational community structure identified above, which was estimated from the inferred live poultry trade network, provides a framework to evaluate AIV dissemination during outbreaks. However, available AIV genome sequence data from China is not obtained in a structured manner at the national level; hence, few or no genomes are available from some provinces. In order to explore further whether a reliance on convenience sampling hinders our understanding of AIV transmission and spread, we utilized a virus gene flow network (GFN) model that is capable of exploring virus transmission paths through both sampled and unsampled locations (*SI Appendix*, Fig. S5). The GFN model generates measures of virus gene flow among locations that can be directly compared with the poultry trade network reported above. We find that the virus gene flow network closely matches the structure of the live poultry trade network (i.e., Fig. 3). In both the live poultry trade network (*SI Appendix*, Table S2) and the gene flow network (*SI Appendix*, Table S3), we identified provinces that acted as hubs (Anhui, Henan, Hubei, and Hunan) by calculating the in-degree and out-degree of nodes. These hubs, located toward the center of the country and linking adjacent communities, may play a significant role in virus transmission among the five region-level communities. However, the structure of the virus gene flow network (*SI Appendix*, Fig. S5*C*) is notably different from that of the poultry egg trade network (*SI Appendix*, Fig. S6).

## Discussion

The prevention and control of avian influenza in China relies critically on an understanding of its mode of geographic spread (23). Here, we undertook a series of analyses that combined AIV gene sequences from poultry in China with data on the live poultry transportation network in the country. Standard phylogeographic analyses revealed evidence that AIV subtypes in domestic poultry have spread between geographically proximate locations (most likely along national highways), and indicated that the level of trade in live poultry is an important contributor to the dissemination of three AIV lineages. We then used geographic modeling of the poultry trade network to identify five live poultry trade communities that represent the large-scale structure of the poultry trade in China, and confirmed the relevance of this structure to AIV spread in China using two complementary approaches.

Community-structured transmission of avian influenza means that viruses are more likely to originate from, and migrate to, other localities that belong to the same community during AIV outbreaks. The spatial scale of the communities we have identified is regional; that is, they are subnational but larger than individual provinces. Prevention and control efforts should therefore take into account this regional structure and aim to disrupt the movement of live poultry between source and sink locations. Further, to prevent potential AIV introduction into unaffected but high-risk localities, it is important to assess the position of potential recipient locations within the community structure (19, 23). For example, previous work described the spread of H7N9 viruses between the Yangtze River Delta region and the Pearl River Delta region (21); our results also predict the possibility of AIV spread from the Pearl River Delta region to western China, and from the Yangtze River Delta region to northern and western China, via domestic poultry transportation. This finding is confirmed by the wider geographic spread of H7N9 in wave V (22).

Although we found evidence for continuous geographic viral spread among poultry populations in China (Fig. 1), few studies of the spread of AIVs by wild birds at the intracontinental scale (i.e., the contribution of migratory flyways) have found an effect of geographic distance (18, 24, 25). This might result from the qualitatively different movement patterns of migratory birds and domestic poultry. Wild birds migrate between wintering and breeding sites, making several stopovers along their flyway where they can transmit AIV to local waterfowl (26), mammals (27), and the environment (28). Locations that do not act as wintering, breeding, or stopover sites have a low chance of virus introduction. For this reason, viral lineage movement via bird migration may exhibit discrete jumps over large distances, as observed for the recent global spread of H5N8 (8). In contrast, domestic poultry is transported along railroads or national highways within a country (17, 29), and therefore AIV dissemination in domestic poultry may follow a more continuous pattern of spatial spread (17, 29). These differences likely reflect the roles played in AIV transmission by different hosts, with domestic poultry spreading AIVs at regional scales and migratory birds facilitating intercontinental dispersal over large distances (30).

It is difficult to exclude the potential impact on AIV dispersal of interspecies transmission between wild bird and domestic poultry populations (Fig. 2). We attempted here to focus on transmission dynamics among poultry by reconstructing interspecies transmission events and removing virus lineages dominated by sequences from wild birds (*SI Appendix*, *Materials and Methods*). However, the success of this approach (*SI Appendix*, Fig. S7) may be affected by the underrepresentation of AIV genetic diversity coming from wild birds. In China, domestic poultry are sampled more intensively than wild birds and samples from wild birds are concentrated in only a few locations, such as Qinghai and Hong Kong. Thus, active surveillance of both domestic poultry and wild birds is needed for a better understanding of AIV dispersal dynamics in the country. We also directly compared the ability of empirical wild bird migration and poultry trade networks in China to explain AIV lineage movement, using Bayesian phylogeography inference with a GLM extension. We found that wild bird migration may be weakly associated with H5N1 virus dissemination in poultry but not associated with the spatial spread of H5N6 and H7N9 subtypes. This is consistent with the observation that most H5N6 and H7N9 strains to date are mainly circulating in domestic poultry, human, and environmental samples in China rather than in wild birds. We acknowledge two additional caveats. First, although we have proved the feasibility of using a gravity model to reconstruct the live poultry trade network and have found a live poultry trade network that could explain the observed gene flow, uncertainties between the gravity model and disease spread (31) still exist and should be addressed in future work. Second, even though we have tried to avoid the limits of multiresolution modularity and have demonstrated community structure robustness (Dataset S1, Fig. 5, and *SI Appendix*, Figs. S8–S10), we note that the detected LPTCs are not unique; that is, there is no unique way to define the best partition of a network.

In conclusion, we report a national-level community structure of the poultry trade network in China and its association with the spread of all three major AIV lineages in poultry in the country. Further quantitative and qualitative insights into the network of the live poultry trade and AIV in China will benefit the development of strategies for AIV prevention and control.

## Materials and Methods

Detailed information describing materials and methods is provided in *SI Appendix*.

### Sequence Data.

We accessed HA gene segment sequences of H5N1 AIVs sampled from 1996 to 2014 from the GenBank database, and obtained HA gene segment sequences for H7N9 and H5N6 AIVs sampled from 2013 to 2017 from the Global Initiative on Sharing All Influenza Data (GISAID) database. In order to focus on virus dissemination among poultry, it was first necessary to exclude from the viral phylogenies those chains of AIV transmission that derive from interspecies transmission events and occur in wild bird populations. This was achieved by reconstructing the movement of AIV lineages between wild birds and domestic poultry using a range of sampling and analysis strategies. AIV sequences belonging to phylogenetic clusters that were determined to represent transmission wholly or predominately in or from wild birds were removed from the datasets (*SI Appendix*, Fig. S7), which means that poultry AIV sequences found within clades dominated by wild bird AIV sequences were also excluded. Consequently, only those lineages determined to be circulating in domestic poultry populations were retained for further analysis. In order to ameliorate potential sampling biases, we subsequently randomly subsampled these datasets in a stratified manner to create a more equitable spatiotemporal distribution of AIV sequences (*SI Appendix*, Fig. S11). A strong phylogenetic temporal structure was detected in all datasets (*SI Appendix*, Fig. S12).

### Phylogeographic Inference.

Time-measured phylogenies were inferred using the Bayesian discrete phylogeographic approach (14) implemented in the Bayesian Evolutionary Analysis Sampling Trees (BEAST) program, version 1.8.2 (32). We used an uncorrelated lognormal (UCLN) relaxed molecular clock model (33), the SRD06 nucleotide substitution model (34), and the Gaussian Markov random field (GMRF) Bayesian skyride coalescent tree prior (35). We used Bayesian stochastic search variable selection (BSSVS) to determine 1) the most probable locations of ancestral nodes in the phylogeny and 2) the history and rates of lineage movement among locations (14). To ensure that the relationships between distance and lineage movement were not a consequence of the prior used, we repeated the analysis after randomizing the locations assigned to each sequence (*SI Appendix*, Fig. S13).

To determine potential explanatory factors associated with AIV dispersal among poultry in different locations, we applied the GLM extension of Bayesian phylogeographic inference (15) to the HA gene datasets of the H5N1, H5N6, and H7N9 viruses in China.

### Poultry Transportation Network Reconstruction.

Province-level networks of poultry transportation (Fig. 3 and *SI Appendix*, Fig. S6) were constructed from statistics of poultry egg production and populations of domestic poultry, using a classic gravity model. This model is supported by cross-sectional surveys of poultry transportation in Cambodia (36) and Vietnam (37). In summary, the flux of live poultry or poultry egg transport (*G*_*ij*_) between provinces *i* and *j* separated by geographic distance *d*_*ij*_ takes the form *G*_*ij*_ *= N*_*i*_*N*_*j*_*d*_*ij*_^−1^, where *N*_*i*_ is the number of live poultry (unit: 10,000 poultry) or the amount of poultry egg production (unit: ton) in source province *i* (averaged across years) and *N*_*j*_ is human population size (unit: 10,000 people) in destination province *j* (averaged across years). Other gravity-model parameterizations were tested, and the results showed that the network structure we determined is robust to the parameterization.

### Community Structure Detection.

Identifying community structures (38) is a crucial step in investigating networks that might explain patterns of viral spatial dissemination. In this context, a community is a group of nodes in a network such that intragroup connections are stronger or more numerous than intergroup connections (39, 40); the degree to which a network is subdivided into communities is measured as modularity (40). Using the Walktrap community-finding algorithm (41), with random walk length *t* = 5, we identified the community structure of 1) the live poultry trade networks and 2) the poultry egg trade network. Several community detection methods were compared (*SI Appendix*, Figs. S14 and S15), and the results indicated that current methods were appropriate for our study.

### Data Availability.

HA gene segment sequences are available in the GenBank (www.ncbi.nlm.nih.gov/genome/viruses/variation/flu/) and GISAID (platform.gisaid.org/) databases (Dataset S2). Socioeconomic data are available in statistical yearbooks (*SI Appendix*, Table S4).

## Supplementary Material

Supplementary File

Supplementary File

Supplementary File

Supplementary File
