# Supplementary material for: Assessing the role of live poultry trade in community-structured transmission of avian influenza in China
Source: Proc Natl Acad Sci U S A. 2020 Mar 2;117(11):5949–54. doi: 10.1073/pnas.1906954117 (PMC7084072; doi:10.1073/pnas.1906954117)
Supplement: Supplementary File [file pnas.1906954117.sapp.pdf]

PNAS
www.pnas.org

Supplementary Information for

Assessing the role of live poultry trade in community-structured transmission of avian influenza in China

Qiqi Yang[a], Xiang Zhao[b], Philippe Lemey[c], Marc A. Suchard[d], Yuhai Bi[e,f], Weifeng Shi[g], Di Liu[h], Wenbao Qi[i], Guogang Zhang[j], Nils Chr. Stenseth[k,1], Oliver G. Pybus[l,m,1], Huaiyu Tian[a,1]

Huaiyu Tian
tianhuaiyu@gmail.com;

Oliver G. Pybus
oliver.pybus@zoo.ox.ac.uk;

Nils Chr. Stenseth
n.c.stenseth@ibv.uio.no

This PDF file includes:

- Supplementary text
- Figs. S1 to S15
- Tables S1 to S4
- Captions for Dataset S1, S2, S3
- References for SI reference citations

Other supplementary materials for this manuscript include the following:

Dataset S1, S2, S3 (separate files)
Nucleotide alignments, BEAST XML files, and scripts for reconstruction of poultry trade network, community detection, and the following robustness test (available at the GitHub repository: https://github.com/kikiyang/AIVPoultryChina)

www.pnas.org/cgi/doi/10.1073/pnas.1906954117

Supplementary Information Text

SI Materials and Methods

Sequence data. We accessed haemagglutinin (HA) gene segment sequences of H5N1 AIVs sampled from 1996 to 2014 from the GenBank database, and obtained HA gene segment sequences for H7N9 and H5N6 AIVs sampled from 2013 to 2017 from the GISAID database. Duplicate entries and recombinant strains identified using RDP4 (1) were removed. From these sequence sets, we retained only those sampled in China and for which there was known information on date and location of sampling. In order to focus on virus dissemination among poultry, it was first necessary to exclude from the viral phylogenies those chains of AIV transmission that derive from interspecies transmission events and which occur in wild bird populations. This was achieved by reconstructing the movement of AIV lineages between wild birds and domestic poultry using a range of sampling and analysis strategies. AIV sequences that belong to phylogenetic clusters that are inferred to represent transmission wholly or predominately in or from wild birds were removed from the data sets (*SI Appendix*, Fig. S7), which means that poultry AIV sequences found within clades dominated by wild bird AIV sequences were also excluded. Consequently, only those lineages that are inferred to be circulating in domestic poultry population were retained for further analysis.

In order to ameliorate potential sampling biases, we subsequently randomly subsampled these datasets in a stratified manner to create a more equitable spatiotemporal distribution of AIV sequences. Specifically, the H5N1 HA gene sequences were subsampled to 322 sequences (1704 nt); sequences from over-sampled years and provinces were removed randomly so that there was at most 8 sequences per year and per province, thereby increasing the evenness of sampling whilst retaining a wide range of sampling dates and locations (*SI Appendix*, Fig. S11). In addition, those locations for which there are too few (<4) H5N1 sequences to be analysed in phylogeographic dynamics were removed. Similarly, HA sequences of H7N9 and H5N6 viruses were subsampled to 291 sequences (444 sequences for the full-host dataset of H7N9) and 201 sequences, respectively (*SI Appendix*, Fig. S11). Accession numbers of all sequences in the final datasets are provided in Dataset *S2*.

Initial maximum likelihood (ML) phylogenies were estimated for each subtype using FastTree v2.1.4 (2) under a GTR+Γ nucleotide substitution model. These phylogenies were then used to test for the presence of phylogenetic temporal structure, by generation of a scatterplot of root-to-tip genetic divergence against date of sampling using TempEst v1.5 (3). Strong phylogenetic temporal structure was detected in all datasets (*SI Appendix*, Fig. S12).

Phylogeographic inference. Time-resolved phylogenies of HA sequences were estimated using the Markov chain Monte Carlo (MCMC) approach implemented in BEAST v1.8.2 (4) with the BEAGLE (5) library. We used a uncorrelated lognormal (UCLN) relaxed molecular clock model (6), the SRD06 nucleotide substitution model (7) and the Gaussian Markov random field (GMRF) Bayesian Skyride coalescent tree prior (8). For each dataset, MCMC chains were run in triplicate for 100 million generations with burn-in of 10%, sampling every 10,000 steps. Convergence of MCMC chains was checked with Tracer v1.7. A set of 1000 trees was subsampled from the MCMC chain and used as an empirical tree distribution for the subsequent analysis.

Time-measured phylogenies were inferred using the Bayesian discrete phylogeographic approach (9) implemented in BEAST v1.8.2 (4). We used a non-reversible discrete-state continuous time Markov chain (CTMC) model and a Bayesian stochastic search variable selection (BSSVS) approach to infer (i) the most probable locations of ancestral nodes in the phylogeny and (ii) the history and rates of lineage movement among locations (9). For each dataset, one MCMC chain was run for 200 million steps with a burn-in of 10% steps, sampled every 20,000 steps. Similarly, we assessed the convergence of the chains in Tracer v1.7. Using TreeAnnotator v1.8.2, we subsequently summarized a maximum clade credibility tree from the posterior set of trees of each dataset. To ensure that the inferred relationships

between distance and lineage movement were not a consequence of the prior used, we repeated the analysis after randomising the locations assigned to each sequence (*SI Appendix*, Fig. S13).

Quantifying contributions of potential predictors of AIV dispersal. To infer potential explanatory factors that are associated with AIV dispersal among poultry in different locations, we applied the generalized linear model (GLM) extension of Bayesian phylogeographic inference (10) to the HA gene datasets of H5N1, H5N6 and H7N9 viruses in China. We used a Bayesian Stochastic Search Variable Selection (BSSVS) approach with binomial prior probability distributions on the indicator variables reflecting a 50% prior probability on no predictors being included. We used Bayes factors (BFs) to quantify the support for the posterior probability of inclusion of each potential predictive factor.

The potential predictors used in the model were (i) among-province poultry trade flux, obtained using from the reconstructed live poultry trade network (outlined above), (ii) egg trade flux among provinces in the reconstructed egg trade network (outlined above), (iii) the shortest distance among provinces along the national highway network (see above), (iv) socio-economic predictors relating to production and consumption of live poultry and poultry egg (described in *SI Appendix*, Table S4), specifically, poultry population density, poultry production, poultry consumption and

per capita consumption, egg production, egg consumption and *per capita* consumption, and demographic data, (v) absolute latitude and longitude of the capital city of each province, (vi) number of sequences from each location, and (vii) the existence of bird migration among provinces. Bird migration flux was ascertained from a binary network of wild bird migration among provinces, based on GPS tracking of wild bird migration routes in China from 2006 to 2016, obtained from the Chinese Academy of Forestry (11). All the data (Dataset S3) were log-transformed and normalized as the model inputs.

Interspecies transmission inference. The host transmission between wild avian hosts and domestic poultry hosts throughout the viral evolutionary history was inferred using a Bayesian discrete phylogeographic approach (9) implemented in BEAST v1.8.2 (4). We implemented a non-reversible discrete-state continuous time Markov chain (CTMC) model and a Bayesian stochastic search variable selection (BSSVS) approach to infer the most probable ancestral host of the phylogeny and the most parsimonious description of the phylogeography dynamics (9). MCMC chains were run for 200 million steps with a burn-in of 10% steps, and sampled every 20,000 steps. The maximum clade credibility tree was summarized by TreeAnnotator v1.8.2. Three datasets with different sampling strategies are used in the inference. The host species of each AIV gene sequence is defined according to the United States Geological

Survey (USGS) (12) and by information in the original references. All three trees have a backbone of domestic poultry states (Supplementary Fig. 5).

Sampling strategies. *Dataset 1:* 175 sequences of domestic poultry and 142 sequences of wild birds. We subsampled the HA gene segments of H5N1 viruses from domestic poultry to 175 sequences (1704 nt), at most 2 sequences per year and per province so that the amount of sequences of domestic poultry is nearly equal to that of wild birds (142 sequences). Sequences of wild birds are retained as 142 sequences without down-sampling.

Dataset 2: 353 sequences of domestic poultry and 142 of wild birds. We subsampled the HA gene segments of H5N1 viruses from domestic poultry to 353 sequences (1704 nt), at most 8 sequences per year and per province. Sequences of wild birds are retained as 142 sequences without down-sampling.

Dataset 3: 285 HA sequences of H5N1 of domestic poultry and 84 of wild birds. Sequences of domestic poultry and wild birds are together randomly subsampled to 5 sequences per year, per host type, per location.

Poultry transportation network reconstruction. Provincial-level networks of poultry transportation (Fig. 3, *SI Appendix*, Fig. S6) were constructed from statistics

of poultry egg production and populations of domestic poultry, using a classic gravity model. This model is supported by cross-sectional surveys of poultry transportation in Cambodia (13) and Vietnam (14). In summary, the flux of live poultry or poultry egg transport (G_{ij}) between provinces *i* and *j* separated by geographic distance d_{ij} takes the form $G_{ij} = N_i N_j d_{ij}^{-1}$, where N_i is the amount of live poultry (unit: 10,000 poultry) or poultry egg production (unit: ton) in source province *i* (averaged across years). N_j is human population size (unit: 10,000 people) in destination province *j* (averaged across years). Both N_i and N_j were obtained from statistical yearbooks and databases (*SI Appendix*, Table S4); d_{ij} is the shortest distance (unit: kilometre) between provinces in the national highway network, calculated by the Origin-Destination Cost Matrix algorithm in ArcGIS v10.2 (ESRI, Redlands, CA, USA). This distance was chosen because inter-provincial live poultry transportation in China mainly occurs via national highways; live poultry transportation on freeways is limited due to high tolls, and on railways due to regulations (15, 16). Additionally, other gravity-model parameterisations were tested and the results showed that the network structure we infer is robust to the parameterisation.

Parameter estimation of gravity model. A model with an exponential distance decay function and parameters fit to a dataset from Cambodia (13, 17) was calculated as a contrast to the live poultry trade network. The framework is as follows:

$$G_{ij} = \theta N_i^{\varepsilon} N_j^{\beta} f\left(d_{ij}\right)$$

$$f(d_{ij}) = e^{-\frac{d_{ij}}{\lambda}} / \lambda^2$$

where $\lambda = 167.456$, $\boldsymbol{\varepsilon} = 0.543$, and $\boldsymbol{\beta} = 0.934$ are fitted parameters with data from a cross-sectional survey on live poultry traders in Cambodia, and θ is a scaling parameter equalling 1. The correlation coefficient between trade flows estimated from this model and from the model with classic parameters is 0.698.

Hidden viral transmission path detection. To describe the process of virus transmission across all provinces, including those for which no AIV sequence data was present in our data sets, we used a previously-published gene flow network model (GFN) (18) to infer an empirically-derived weighted network of AIV dispersal (*SI Appendix*, Fig. S5). The GFN model is based on the observation that the pairwise genetic distance between a pair of AIV sequences from two different locations is strongly correlated with the duration of the transmission history between the two infections, which itself is a consequence of the path taken by that history through a given network of locations, whose connections are weighted by some measure (e.g. distance, or trade). A summary of the steps of the GFN model approach follows here. (i) To begin we construct a fully-adjacent network, whose nodes are the geographic locations/provinces (regardless of whether AIV sequences are available for that location) and whose edges represent the minimum distance along national highways between adjacent locations (edges between non-adjacent locations are not excluded). (ii) A hypothesised path through this fully-adjacent network is then proposed. The

path must pass through all locations for which virus sequences are available in a sequential random order; in doing so, the path may pass through locations without virus sampling and may visit the same node more than once. The shortest path meeting these conditions was identified using the Floyd-Warshell Algorithm (19). (iii) A pair of sequences from two locations is chosen. (iv) The spatial distance along the hypothesised path between the locations defined by these two sequences is then calculated. This is simply the sum of the edges in the fully-adjacent network as the path moves through it. (v) Steps (ii)-(iv) are then repeated for all pairs of sequences. The correlation between the genetic distance and path spatial distance for each pair of sequences is then computed. (vi) Steps (ii) to (v) are then repeated 10,000 times, resulting in a distribution of correlation coefficient values. (vi) The top 10% of these replicates with the highest correlation coefficients are identified. (vii) The hypothesised paths that correspond to the top 1000 paths are then summarised into the final gene flow network (GFN). This is done by simply counting the frequency with which each edge in the fully-adjacent network is represented in the set of 1000 paths. These frequencies then reflect the likelihood of virus movement among all locations, including via locations for which no sequences were sampled.

Community structure detection. Identifying community structures (20) is a crucial step in investigating networks that might explain patterns of viral spatial dissemination. In this context, a “community” is a group of nodes in a network such

that intra-group connections are stronger or more numerous than inter-group connections (21, 22); the degree to which a network is subdivided into communities is measured as “modularity” (22). Using the Walktrap community finding algorithm (23), with random walk length $t = 5$, we identified the community structure of (i) the live poultry trade networks, (ii) the poultry egg trade network. Although each of these networks are directed, edge directions were ignored in order to focus on vertex connections. The connection strength of two vertices was defined as the sum of the weights of all edges between those vertices; this approach has proven suitable for modularity-based community detection algorithms (20, 22).

To set the random walk length t to a suitable value, we experimented the community structure detection with t from 1 to 100. We observed that small values like t=1, 2, 3, 4, 5 generated community structures with much higher modularity, which is consistent with the previous study (24). When t=2, the modularity value is highest, whereas locations are merged into only two large communities, which probably results from the resolution limit of modularity optimization (25). To sufficiently capture the local community structure and avoid over optimization of modularity as well, we set the random walk length t to 5.

To test the robustness of the detected community structures, we introduced the random perturbation as Gaussian noise (mean=0, standard deviation=10% of the mean

edge weights) into the live poultry network. On the perturbed networks, we conducted the community structure detection by Walktrap community finding algorithm, with random walk length *t*=5. To quantify differences in community structure of the perturbed network and that of the original network, we used four representative methods including the Rand index (26), van Dongen metric (27), normalized mutual information (28) and variation of information (29). The results (Dataset S1) show that the detected community structures are stable.

References

1. D. P. Martin, B. Murrell, M. Golden, A. Khoosal, B. Muhire, RDP4: Detection and analysis of recombination patterns in virus genomes. *Virus Evol* **1**, vev003 (2015).
2. M. N. Price, P. S. Dehal, A. P. Arkin, FastTree: computing large minimum evolution trees with profiles instead of a distance matrix. *Mol Biol Evol* **26**, 1641-1650 (2009).
3. A. Rambaut, T. T. Lam, L. M. Carvalho, O. G. Pybus, Exploring the temporal structure of heterochronous sequences using TempEst (formerly Path-O-Gen). *Virus Evol* **2**, vew007 (2016).
4. A. J. Drummond, M. A. Suchard, D. Xie, A. Rambaut, Bayesian phylogenetics with BEAUti and the BEAST 1.7. *Mol Biol Evol* **29**, 1969-1973 (2012).
5. D. L. Ayres *et al.*, BEAGLE: an application programming interface and high-performance computing library for statistical phylogenetics. *Syst Biol*, syr100 (2011).
6. A. J. Drummond, S. Y. Ho, M. J. Phillips, A. Rambaut, Relaxed phylogenetics and dating with confidence. *PLoS Biol* **4**, e88 (2006).
7. B. Shapiro, A. Rambaut, A. J. Drummond, Choosing Appropriate Substitution Models for the Phylogenetic Analysis of Protein-Coding Sequences. *Mol Biol Evol* **23**, 7-9 (2005).
8. V. N. Minin, E. W. Bloomquist, M. A. Suchard, Smooth skyride through a rough skyline: Bayesian coalescent-based inference of population dynamics. *Mol Biol Evol* **25**, 1459-1471 (2008).
9. P. Lemey, A. Rambaut, A. J. Drummond, M. A. Suchard, Bayesian phylogeography finds its roots. *PLoS Comput Biol* **5**, e1000520 (2009).

10. P. Lemey *et al.*, Unifying viral genetics and human transportation data to predict the global transmission dynamics of human influenza H3N2. *PLoS Pathog* **10**, e1003932 (2014).
11. S. Li *et al.*, Migratory Whooper Swans Cygnus cygnus Transmit H5N1 Virus between China and Mongolia: Combination Evidence from Satellite Tracking and Phylogenetics Analysis. *Sci Rep* 10.1038/s41598-018-25291-1 (2018).
12. United States Geological Survey, List of Species Affected by H5N1 (Avian Influenza). United States Geological Survey. Available at https://www.nwhc.usgs.gov/disease_information/avian_influenza/affected_species_chart.jsp. Deposited 2011.
13. M. D. Van Kerkhove, H5N1/highly pathogenic avian influenza in Cambodia : evaluating poultry movement and the extent of interaction between poultry and humans. PhD thesis, London School of Hygiene & Tropical Medicine. http://dx.doi.org/10.17037/PUBS.00682389 (2009).
14. G. Fournié *et al.*, Investigating poultry trade patterns to guide avian influenza surveillance and control: a case study in Vietnam. *Sci Rep* **6**, 29463 (2016).
15. K. Bingsheng, H. Yijun, "Poultry sector in China: structural changes during the past decade and future trends" in Poultry in the 21st Century: avian influenza and beyond. Proceedings of the International Poultry Conference, held 5–7 November 2007, Bangkok, Thailand., O. Thieme, D. Pilling, Eds. (FAO, Rome, 2008), pp. 85-117.
16. L. Fang *et al.*, Environmental factors contributing to the spread of H5N1 avian influenza in mainland China. *PLoS One* **3**, e2268 (2008).
17. M. D. Van Kerkhove, "Poultry Movement and Sustained HPAI Risk in Cambodia" in Health and animal agriculture in developing countries, D. Zilberman, J. Otte, D. Roland-Holst, D. Pfeiffer, Eds. (Springer, 2011), https://doi.org/10.1007/978-1-4419-7077-0, pp. 233-263.
18. H. Tian *et al.*, Avian influenza H5N1 viral and bird migration networks in Asia. *Proc Natl Acad Sci USA*, doi: 10.1073/pnas.1405216112 (2014).
19. R. W. Floyd, Algorithm 97: shortest path. *Commun ACM* **5**, 345 (1962).
20. M. Girvan, M. E. Newman, Community structure in social and biological networks. *Proc Natl Acad Sci USA* **99**, 7821-7826 (2002).
21. F. Radicchi, C. Castellano, F. Cecconi, V. Loreto, D. Parisi, Defining and identifying communities in networks. *Proc Natl Acad Sci USA* **101**, 2658-2663 (2004).
22. M. E. Newman, M. Girvan, Finding and evaluating community structure in networks. *Phys Rev E* **69**, 026113 (2004).
23. P. Pons, M. Latapy, Computing communities in large networks using random walks. *J. Graph Algorithms Appl.* **10**, 191-218 (2006).
24. D. Lai, H. Lu, C. Nardini, Enhanced modularity-based community detection by random walk network preprocessing. *Phys Rev E* **81**, 066118 (2010).

25. S. Fortunato, M. Barthélemy, Resolution limit in community detection. *Proc Natl Acad Sci USA* **104**, 36-41 (2007).
26. L. Hubert, P. Arabie, Comparing partitions. *J Classif* **2**, 193-218 (1985).
27. S. van Dongen, Performance criteria for graph clustering and Markov cluster experiments. Technical Report INS-R0012, National Research Institute for Mathematics and Computer Science in the Netherlands, Amsterdam (2000).
28. D. Leon, D.-G. Albert, D. Jordi, A. Alex, Comparing community structure identification. *J Stat Mech* **2005**, P09008 (2005).
29. M. Meilă, Comparing clusterings—an information based distance. *J Multivar Anal* **98**, 873-895 (2007).

326 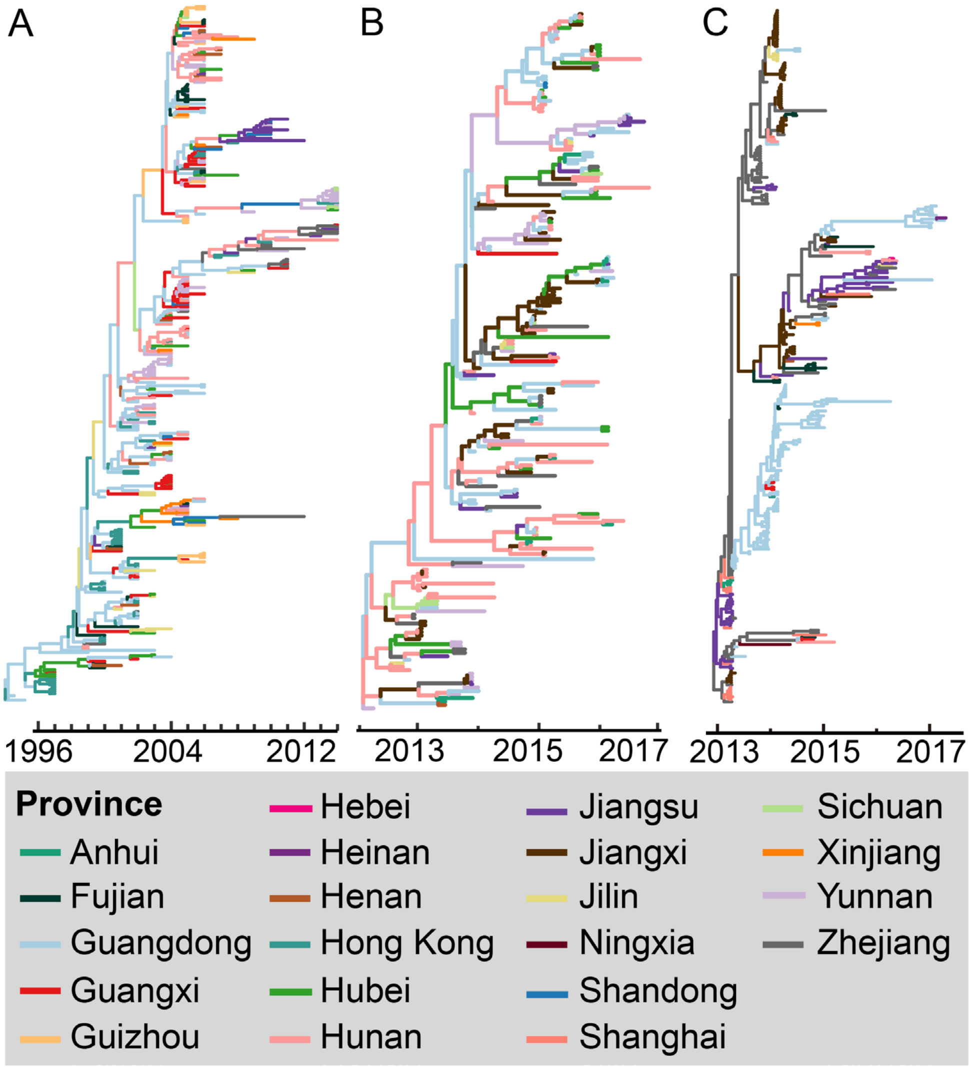

327 **Fig. S1. The maximum clade credibility (MCC) phylogeny of the HA gene of**
328 **H5N1 (A), H5N6 (B) and H7N9 (C) viruses in poultry in China.** The phylogeny is
329 inferred by Bayesian phylogeography inference methods. Branches are coloured
330 according to the most probable location state of their descendent nodes.

331

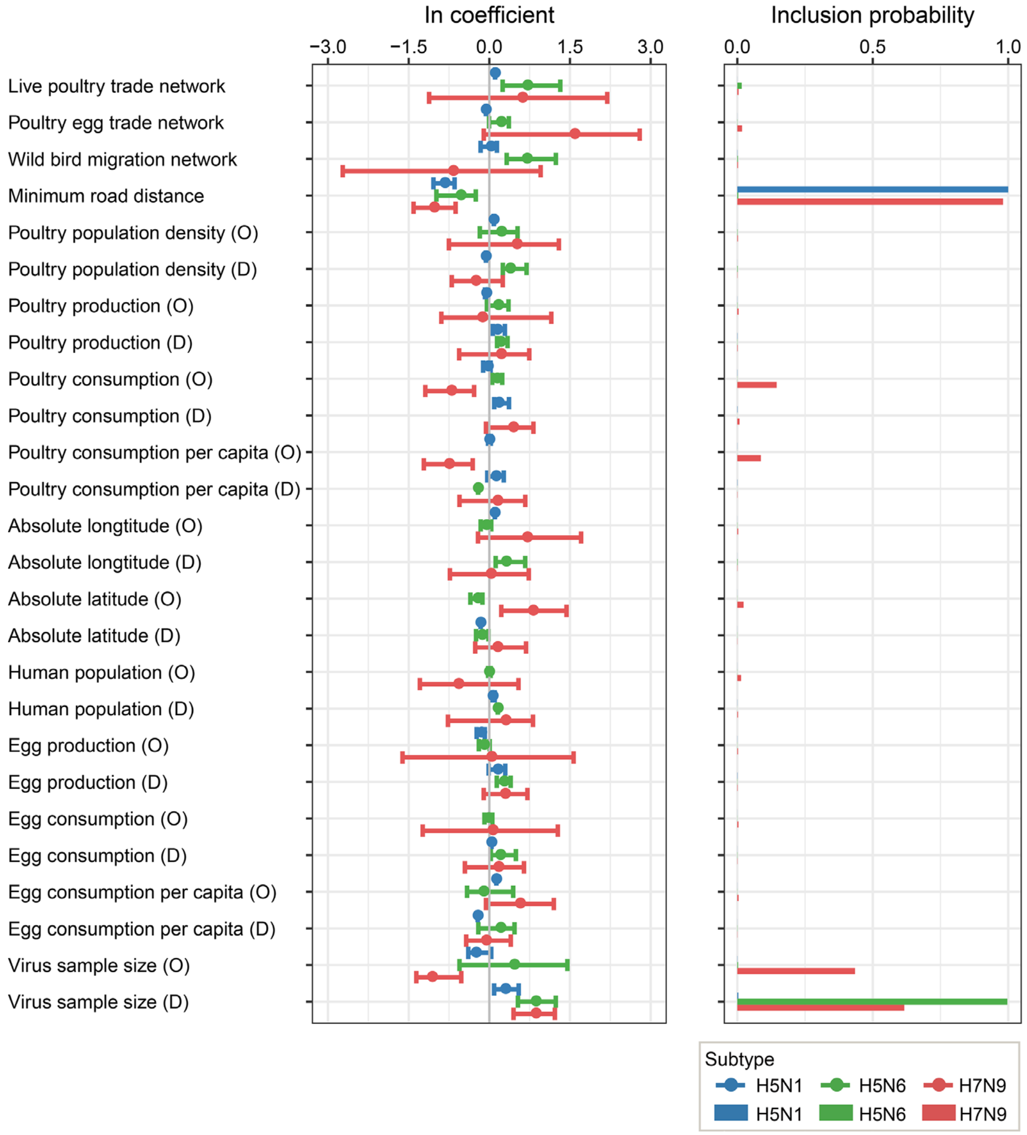

Fig. S2. Contributions of all predictor variables to the dissemination of H5N1, H5N6 and H7N9 lineages in China among poultry, inferred from analysis of HA gene sequences. HP AIV subtypes H5N1, H5N6 and H7N9 are coloured blue, green and red, respectively. Predictors labelled (O) and (D) represent the origin and destination, respectively. In the left-hand plots, the estimated coefficients of predictors are represented as circles (>0 = positive association, <0 = negative association). Error bars represent the 95% highest posterior density (HPD) credible interval for these estimates. The bars in the right-hand plots show the posterior probability of inclusion of each predictor.

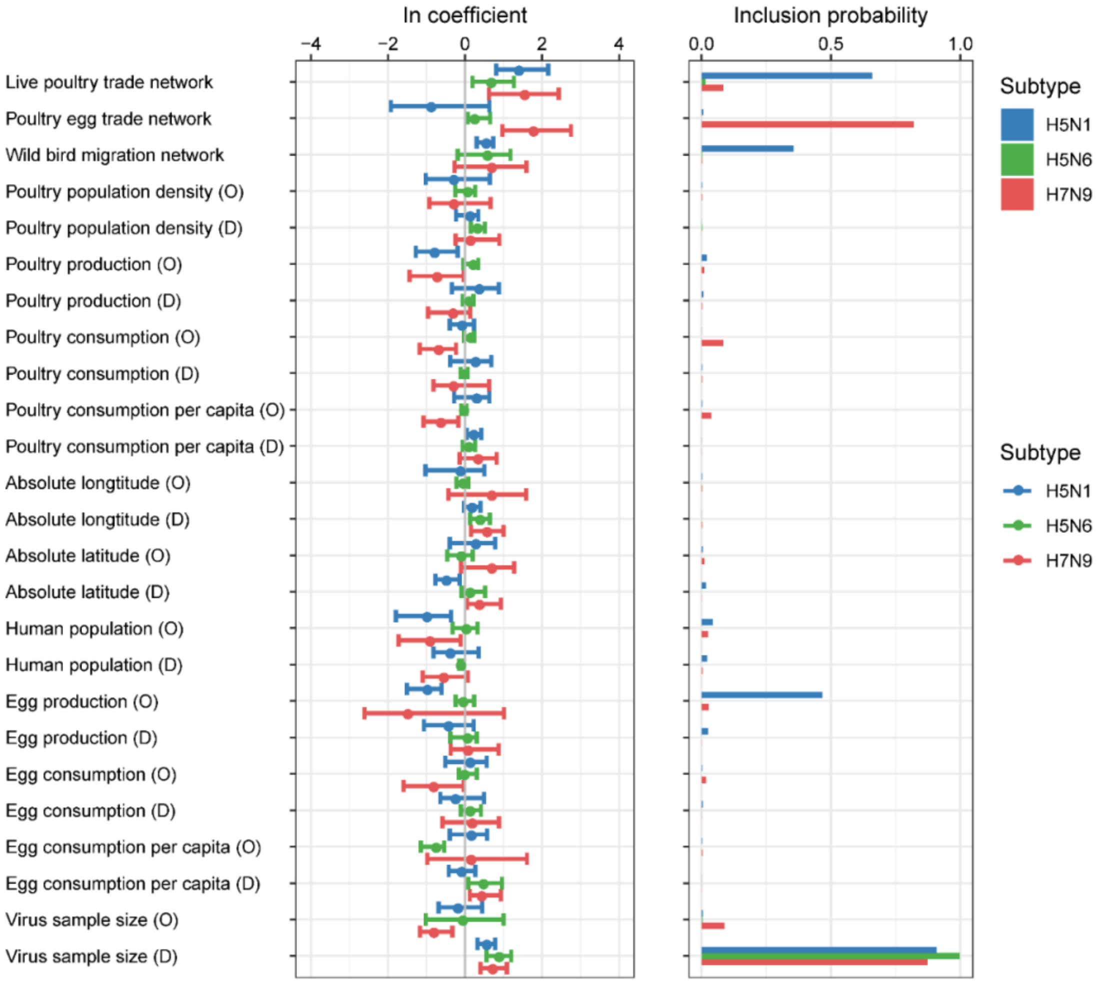

Fig. S3. Contributions of predictor variables to the dissemination of H5N1, H5N6 and H7N9 lineages among poultry in China, inferred from analysis of HA gene sequences, without minimum road network distance. Results when the minimum road network distance predictor is excluded from the analysis. Due to the high similarity of poultry trade and egg trade (R=0.95, *P*<0.01), we do not further differentiate between those two predictors.

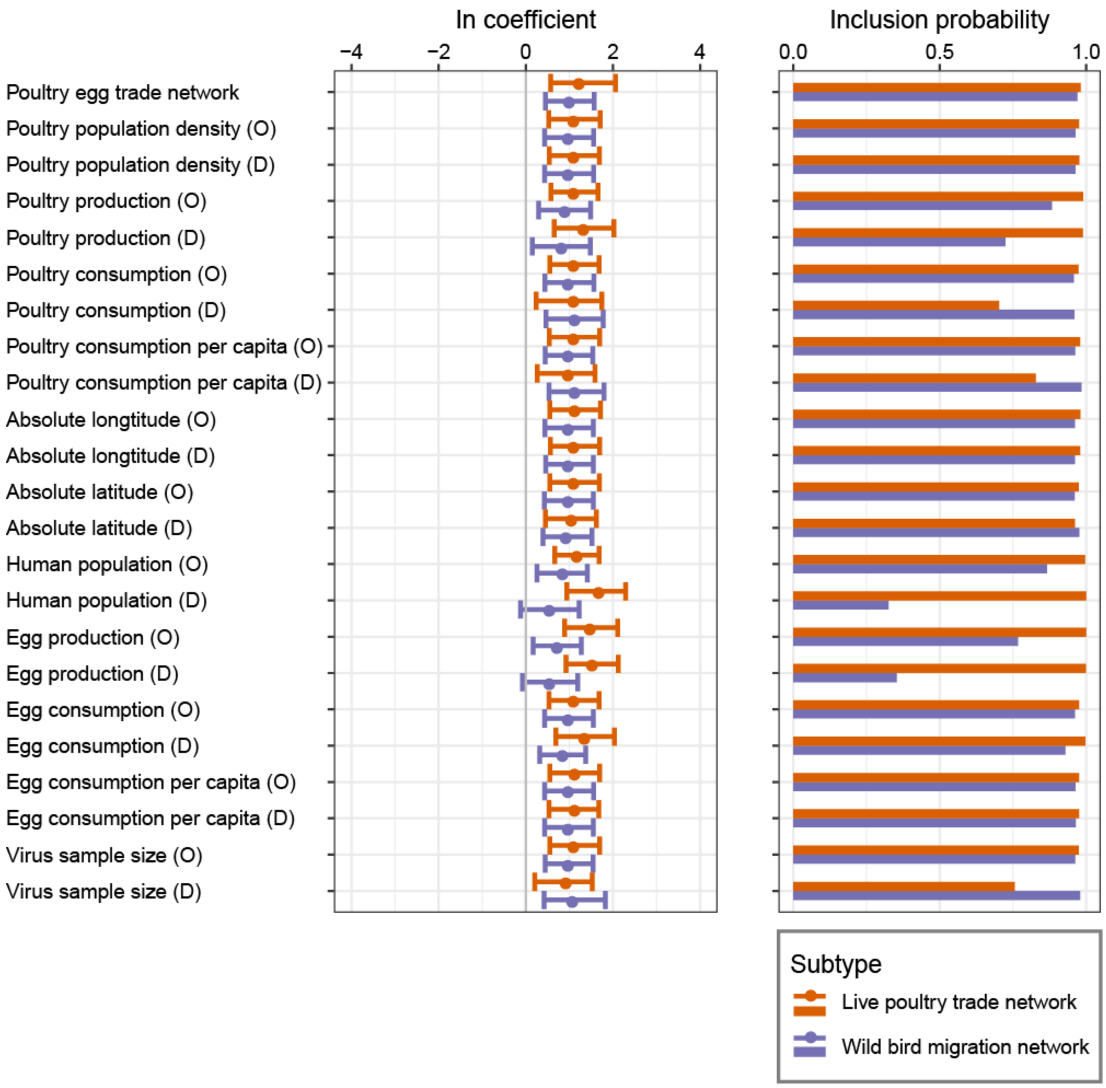

Fig. S4a. Sensitivity analysis, H5N1 AIV. Results show the effect of the inclusion of the third predictor on the estimates of wild bird migration and live poultry trade network.

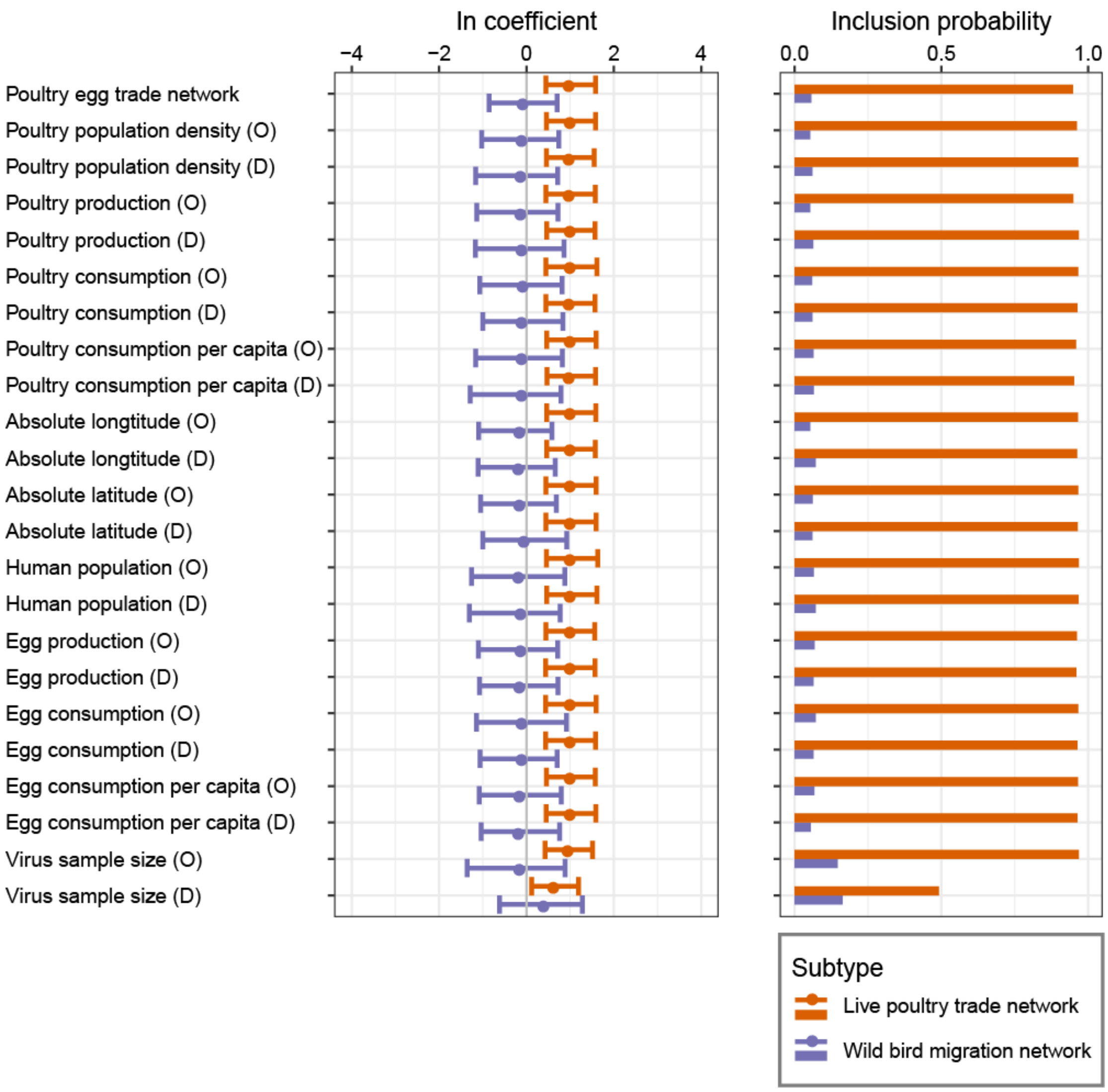

Fig. S4b. Sensitivity analysis, H5N6 AIV. Results show the effect of the inclusion of the third predictor on the estimates of wild bird migration and live poultry trade network.

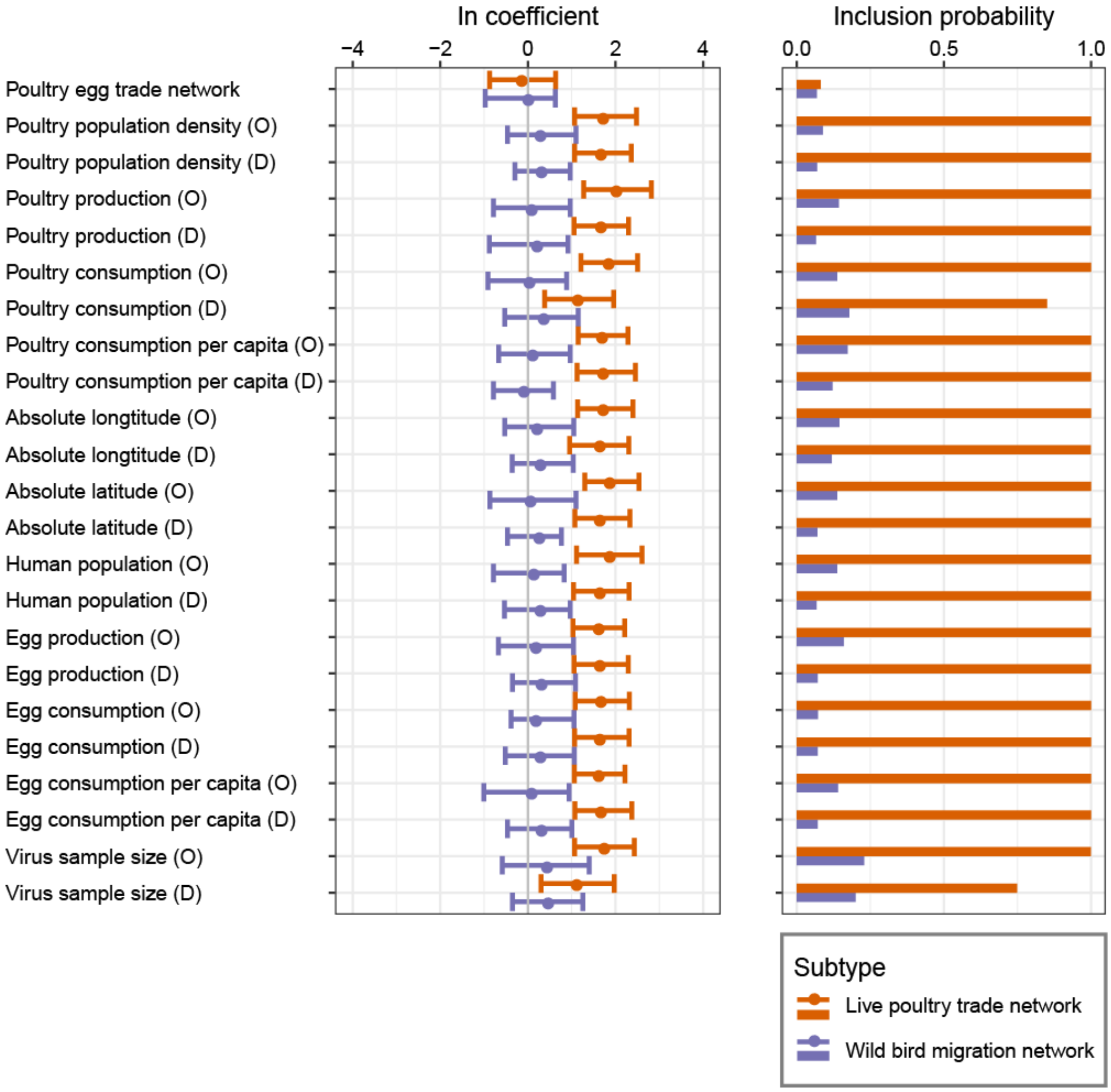

Fig. S4c. Sensitivity analysis, H7N9 AIV. Results show the effect of the inclusion of the third predictor on the estimates of wild bird migration and live poultry trade network. Due to the high similarity of poultry trade and egg trade (R=0.95, P<0.01), we do not further differentiate between those two predictors.

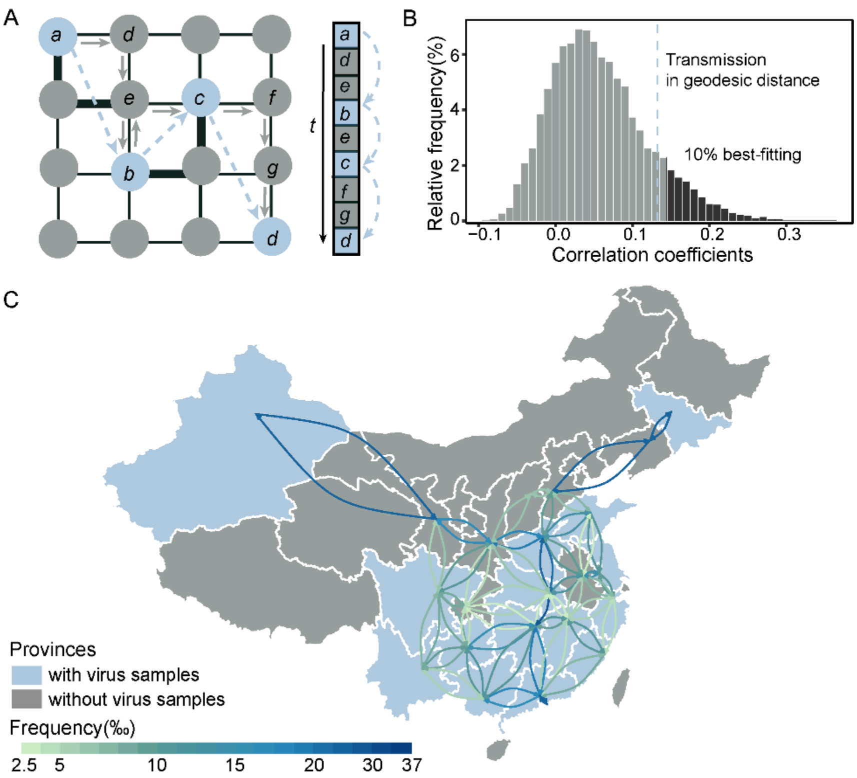

Fig. S5. The gene flow network model. (A) Method of finding the virus transmission path (grey arrow-headed solid lines) in time order (t): a->d->e->b->e->c->f->g->d that is hidden in the full adjacent network under the observed path (blue arrow-headed dotted lines): a->b->c->d. Blue nodes indicate locations from which virus sequences are available; grey indicates locations without virus sampling. Black solid lines between nodes represent edges between adjacent locations in the full adjacent network. Line widths represent edge weights that are used to find the shortest path by Floyd-Warshell algorithm. (B) Histogram of the correlation coefficients of the path distances through the network and the gene flow network of sequences. Bars represent the correlation coefficients generated by 10,000 hypothesised transmission paths and genetic distances, 1000 best-fittings were highlighted in black. The blue dotted line indicates the strength of the correlation obtained if the distance used is simply the geodesic distance between pairs of provinces. (C) Gene flow network. Frequency of migration events between pairs of provinces in 1000 top-selected randomly generated transmission paths. Green and dark blue curves represent low and high frequencies, respectively. Blue indicates provinces from which virus sequences are available; grey indicates provinces without virus sampling.

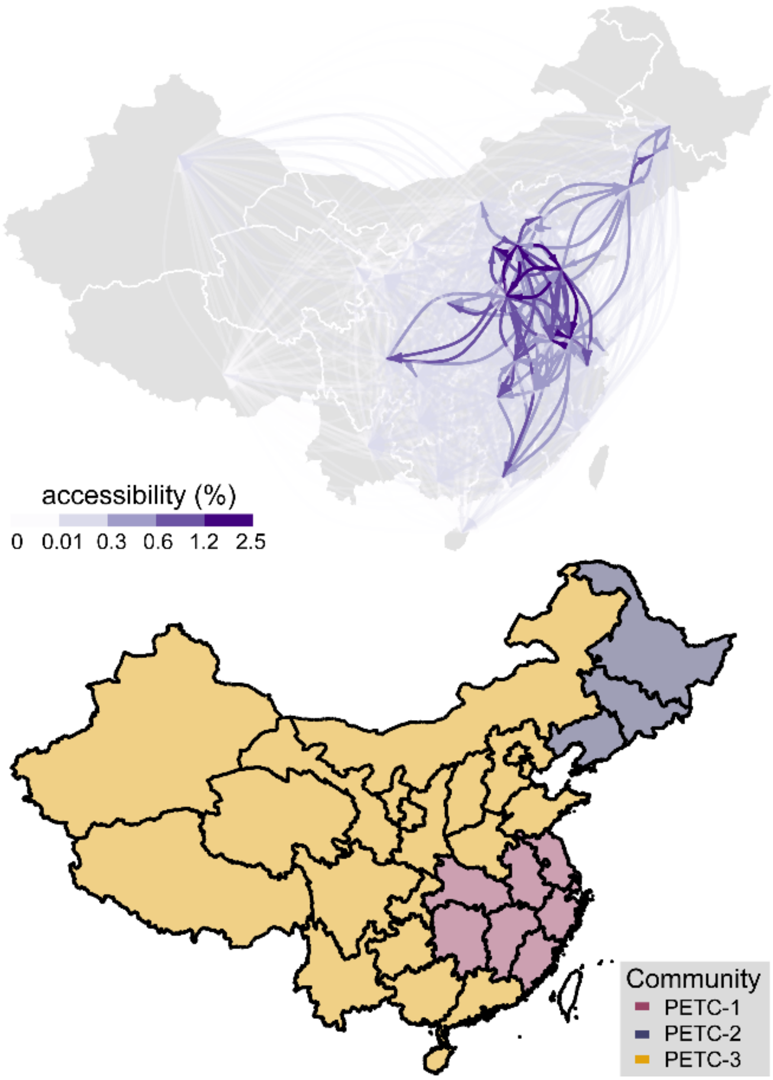

Fig. S6. Poultry egg trade network and community structure. Upper panel: Accessibility of poultry egg trade flows between pairs of provinces. Colours of light purple and dark purple represent low and high accessibility respectively. Lower panel: poultry egg trade communities 1, 2, 3 (coloured pink, purple, and yellow, respectively) are clustered in the Yangtze River Delta region, north-eastern China and other regions.

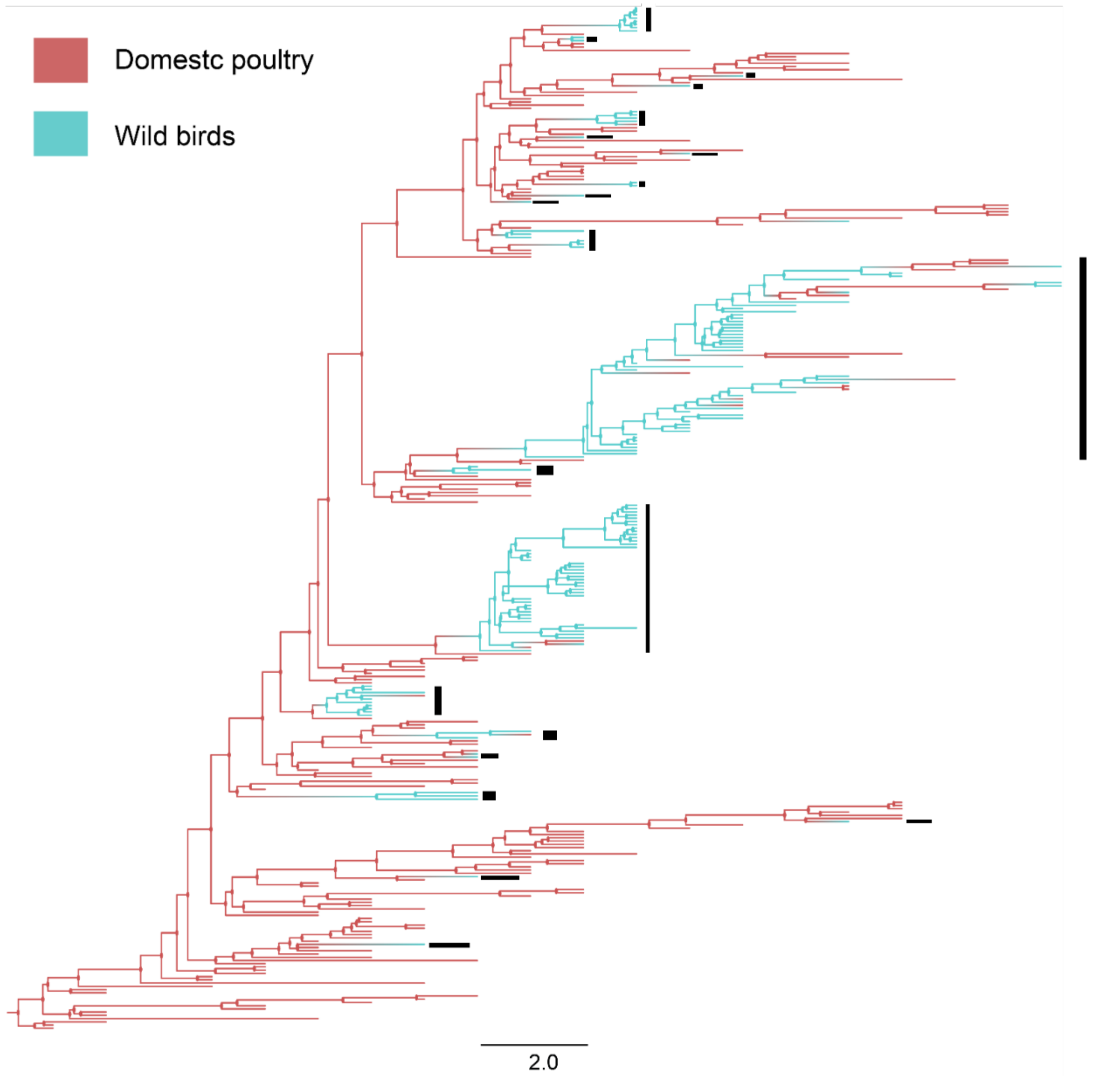

Fig. S7a. The maximum clade credibility (MCC) phylogeny of the HA gene of H5N1 viruses in wild birds and domestic poultry in China inferred by Bayesian discrete phylogeographic approach. Light blue and light red represent the host type of wild birds and domestic poultry, respectively. The phylogeny is inferred on *Dataset 1* (sequences of domestic poultry: 175 and those of wild birds: 142). The sequences indicated by a black rectangle are in the wild bird lineage that would be removed in subsequent analysis.

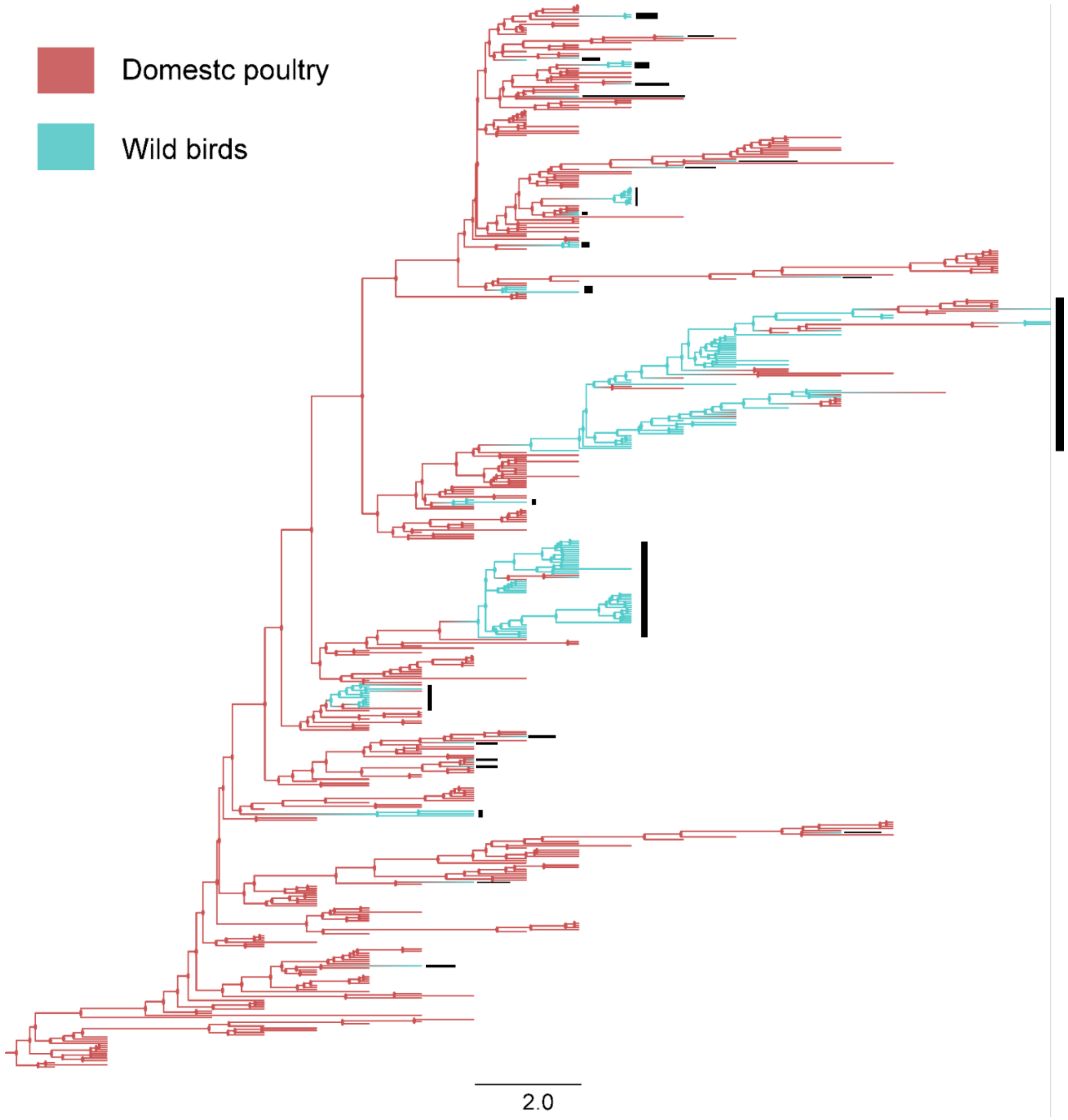

Fig. S7b. The maximum clade credibility (MCC) phylogeny of the HA gene of H5N1 viruses in wild birds and domestic poultry in China inferred by Bayesian discrete phylogeographic approach. Light blue and light red represent the host type of wild birds and domestic poultry, respectively. The phylogeny is inferred on Dataset 2 (sequences of domestic poultry: 353 and those of wild birds: 142). The sequences indicated by a black rectangle are in the wild bird lineage that would be removed in subsequent analysis.

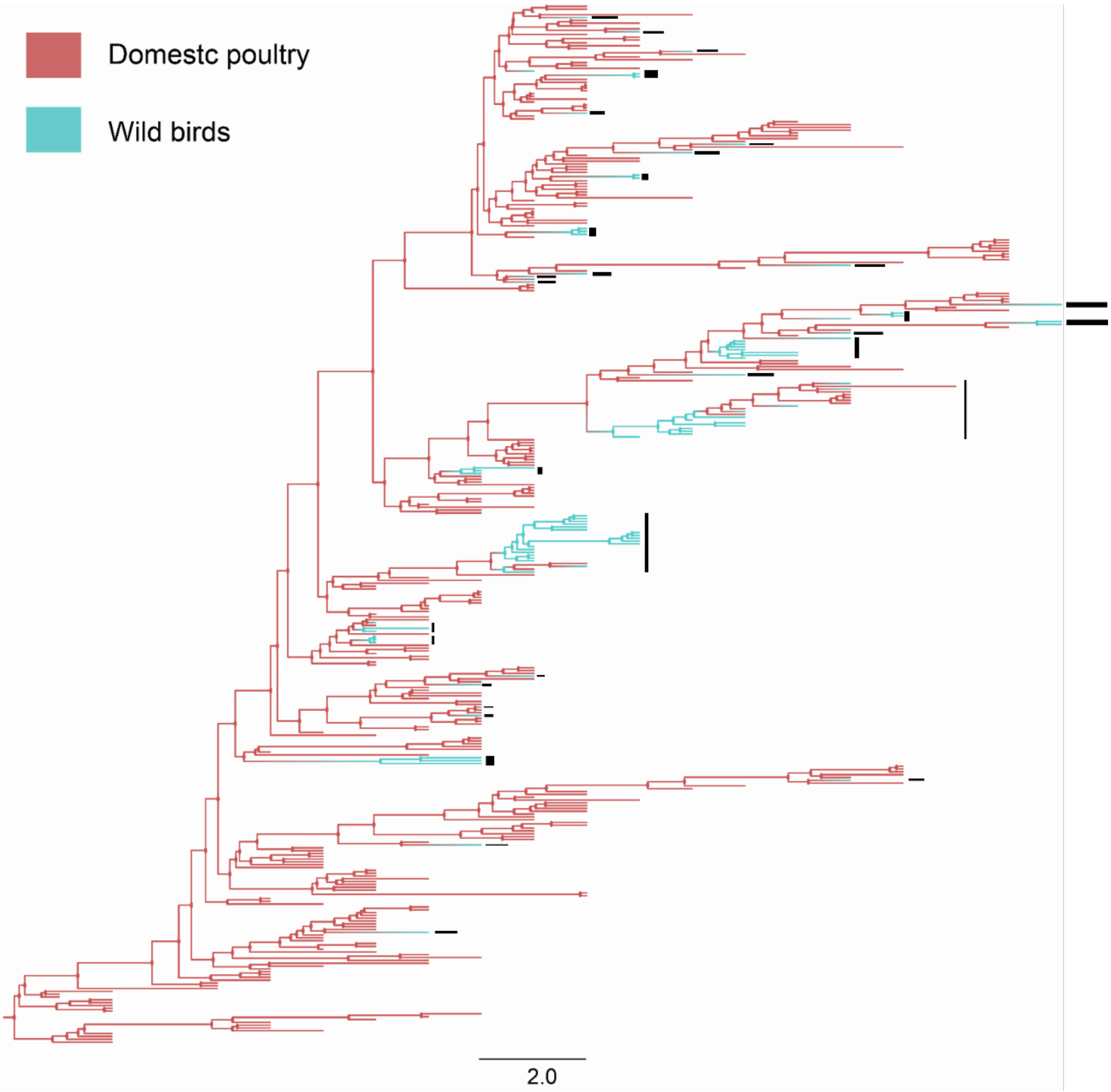

Fig. S7c. The maximum clade credibility (MCC) phylogeny of the HA gene of H5N1 viruses in wild birds and domestic poultry in China inferred by Bayesian discrete phylogeographic approach. Light blue and light red represent the host type of wild birds and domestic poultry, respectively. The phylogeny is inferred on Dataset 3 (sequences of domestic poultry: 285 and those of wild birds: 84). The sequences indicated by a black rectangle are in the wild bird lineage that would be removed in subsequent analysis.

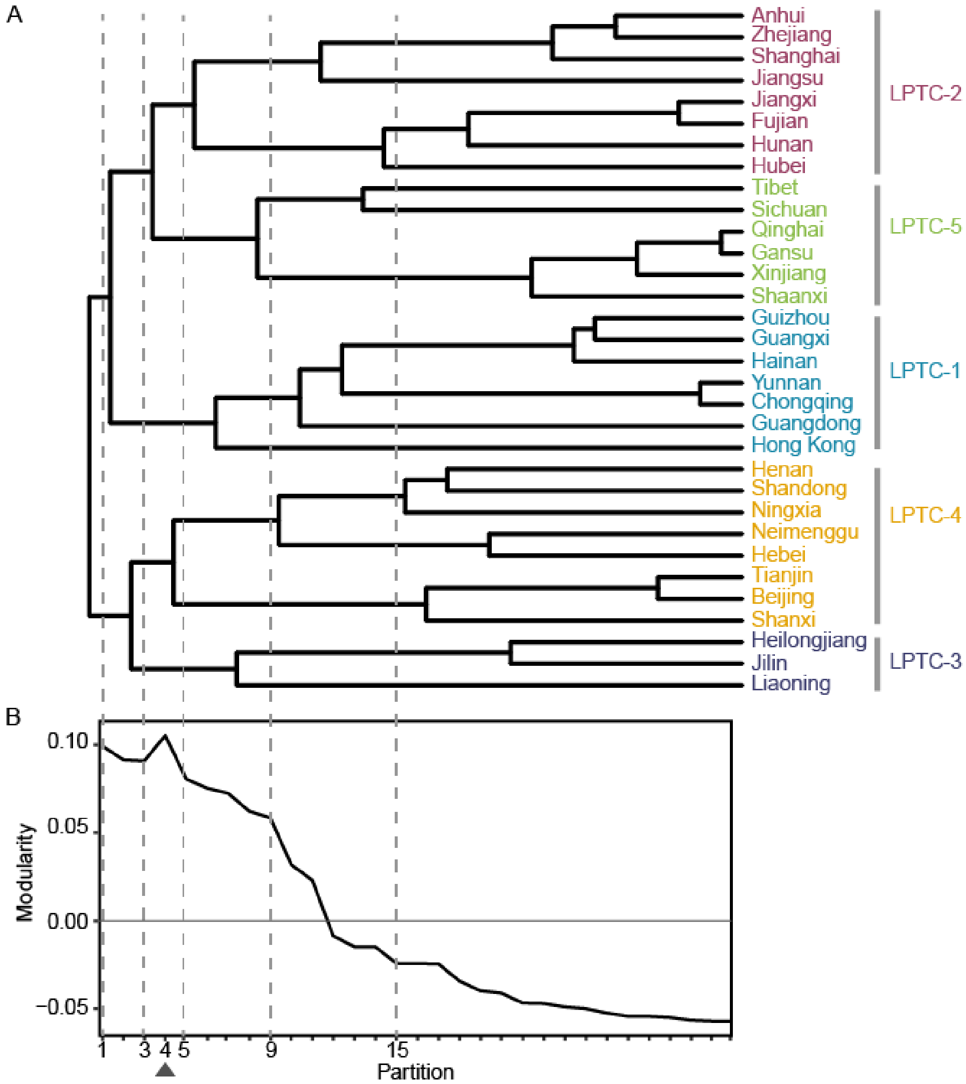

Fig. S8. Community detection at different scales. (A) Multi-scale partitions of the live poultry trade network found by Walktrap algorithm, and (B) the corresponding modularity value at various resolution levels. The triangle indicates the live poultry trade communities (Partition 4) used in the main text. Four community structures at different scales (dotted lines) were selected for comparison. Positive value of the modularity indicates the possible presence of community structure.

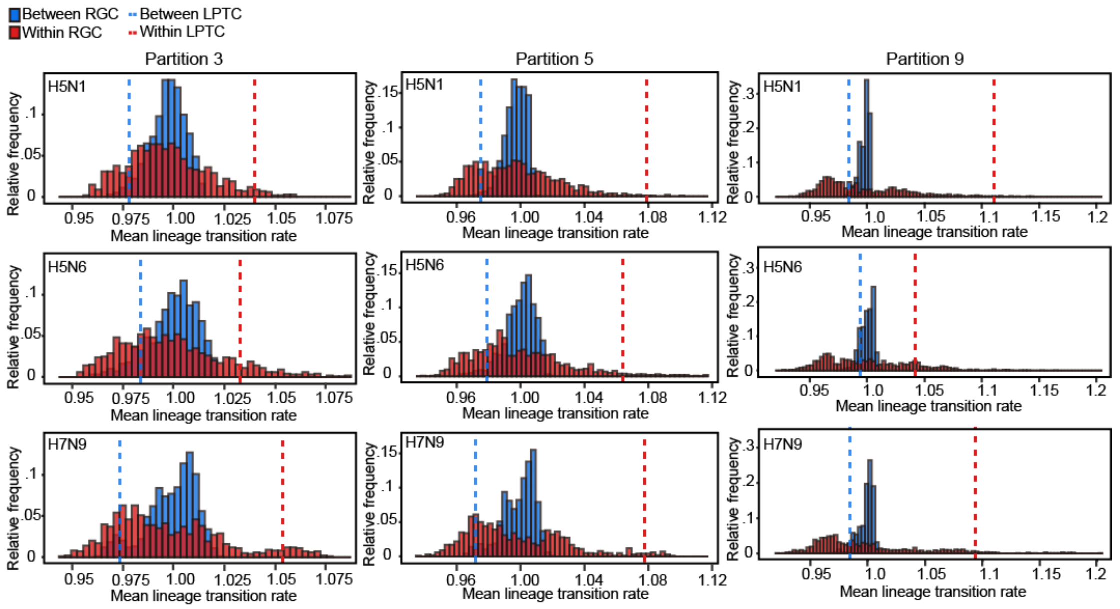

Fig. S9. Histograms of mean among-location AIV lineage transition rates (Partition 3, 5, and 9). The raw transition rates between locations are obtained from the analyses reported in Fig. 1. Two means are shown here: blue = mean transition rate for pairs of locations in different live poultry trade communities (LPTCs); red = mean transition rate for pairs of locations in the same LPTC. Dotted vertical lines show these two means calculated using the partition of the empirically-derived (“true”) LPTC network.

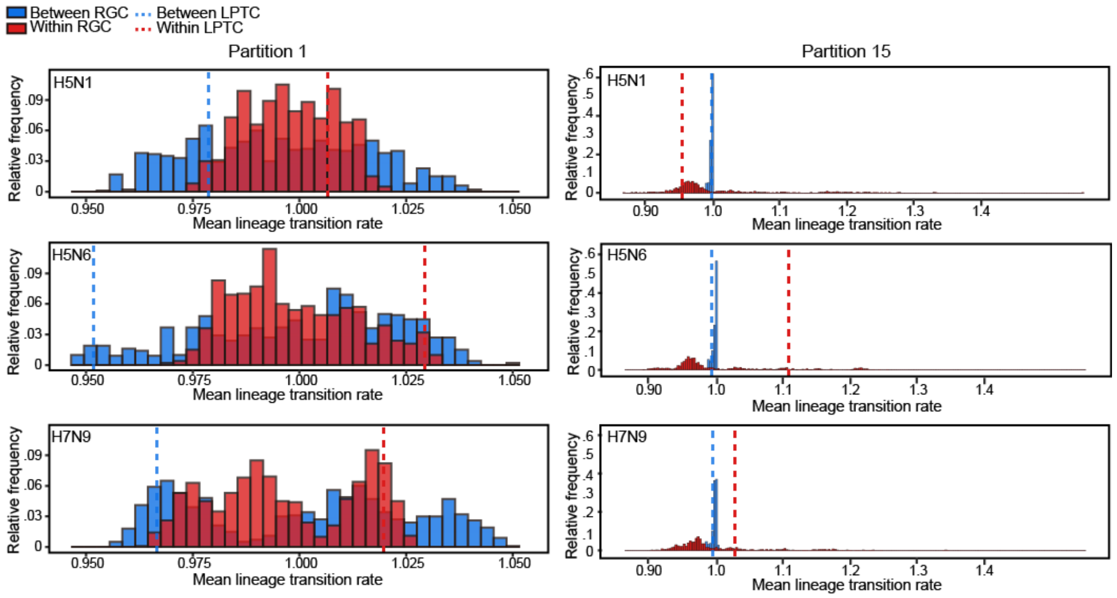

Fig. S10. Histograms of mean among-location AIV lineage transition rates (Partition 1 and 15). The raw transition rates between locations are obtained from the analyses reported in Fig. 1. Two means are shown here: blue = mean transition rate for pairs of locations in different live poultry trade communities (LPTCs); red = mean transition rate for pairs of locations in the same LPTC. Dotted vertical lines show these two means calculated using the partition of the empirically-derived (“true”) LPTC network.

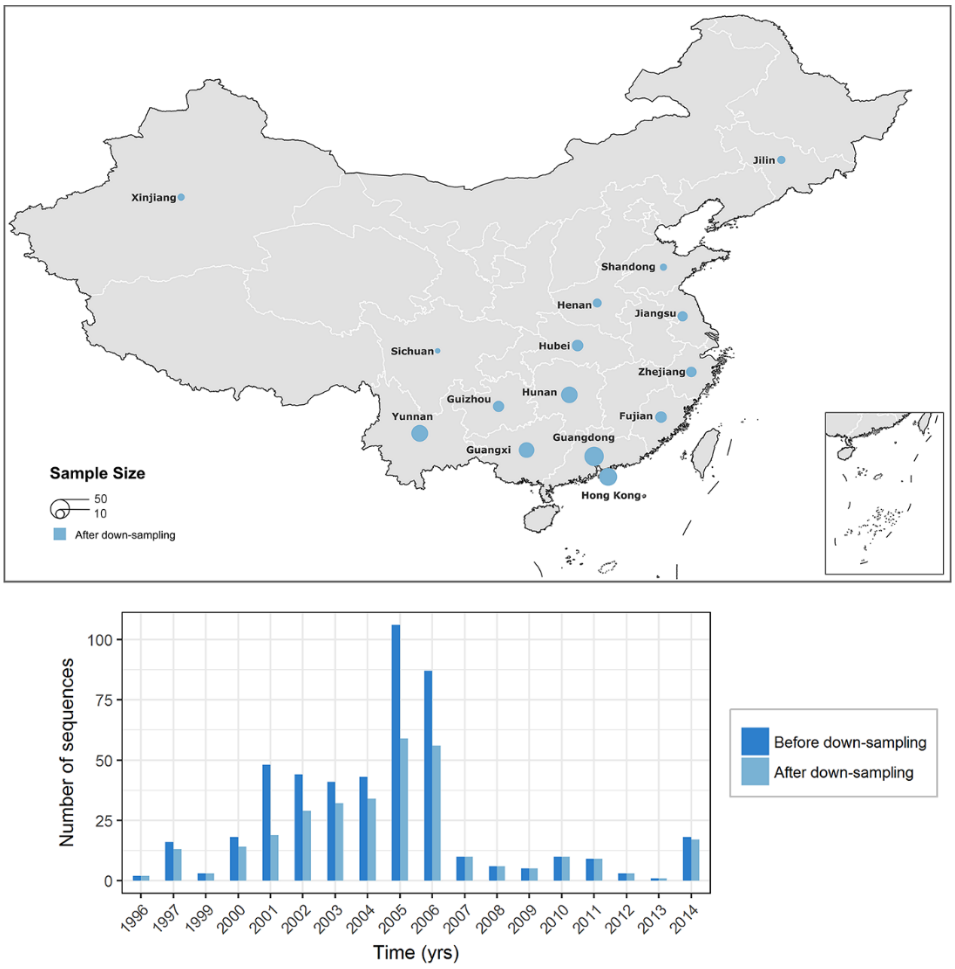

Fig. S11a. Spatial and Temporal distribution of HA sequences of H5N1 viruses used in this study before/after down-sampling. Upper panel: Spatial distribution of HA sequences from domestic poultry between provinces in China after down-sampling. Pie size represents the number of sequences. Lower panel: Temporal distribution of HA sequences from domestic poultry from 1996 to 2017. The dark bar represents sequences before down-sampling and the light bars represent down-sampled sequences.

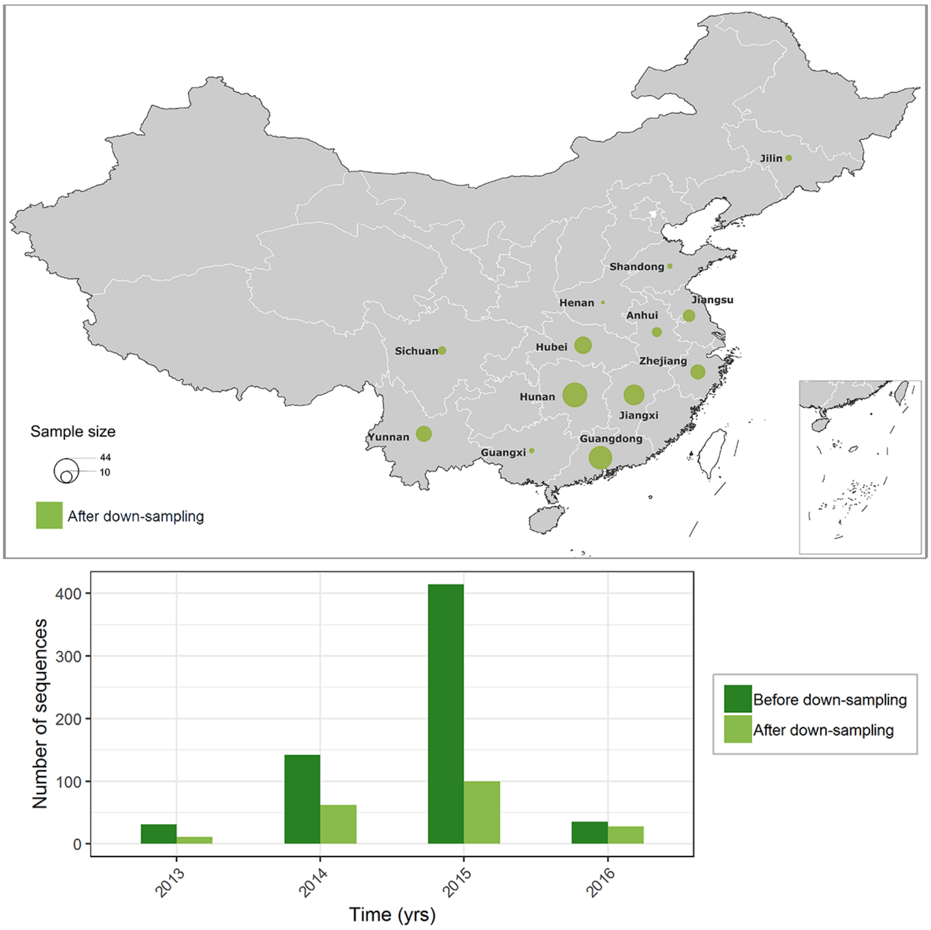

Fig. S11b. Spatial and Temporal distribution of HA sequences of H5N6 viruses used in this study before/after down-sampling. Upper panel: Spatial distribution of HA sequences from domestic poultry between provinces in China after down-sampling. Pie size represents the number of sequences. Lower panel: Temporal distribution of HA sequences from domestic poultry from 2013 to 2018. The dark bar represents sequences before down-sampling and the light bars represent down-sampled sequences.

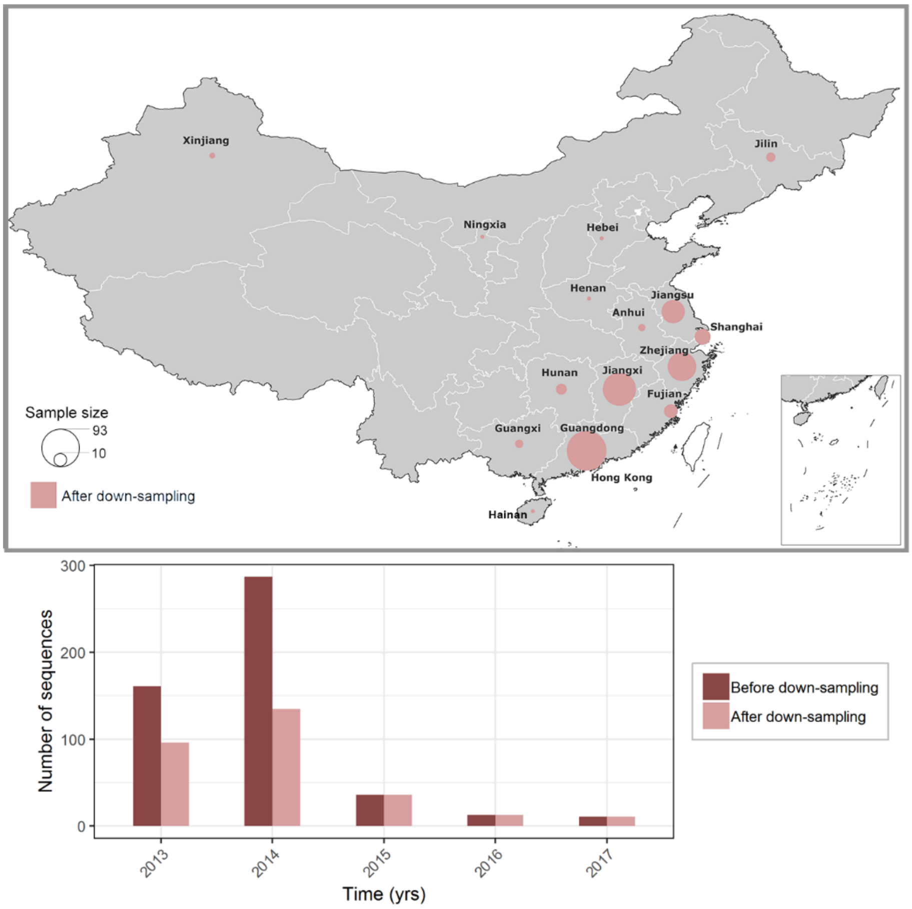

Fig. S11c. Spatial and Temporal distribution of HA sequences of H7N9 viruses used in this study before/after down-sampling. Upper panel: Spatial distribution of HA sequences from domestic poultry between provinces in China after down-sampling. Pie size represents the number of sequences. Lower panel: Temporal distribution of HA sequences from domestic poultry from 2013 to 2017. The dark bar represents sequences before down-sampling and the light bars represent down-sampled sequences.

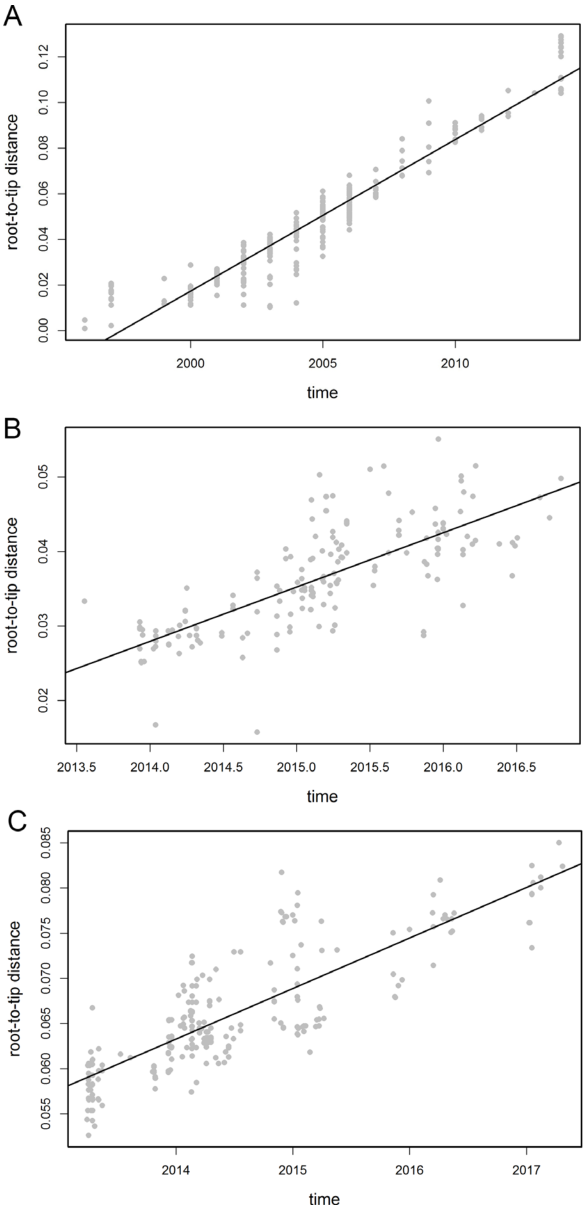

Fig. S12. Strong temporal signal tested in TempEst of H5N1 (A), H5N6 (B) and H7N9 (C). We used the subsampled dataset of sequences of the HA gene segments of each subtype of viruses isolated from domestic poultry in China in this test.

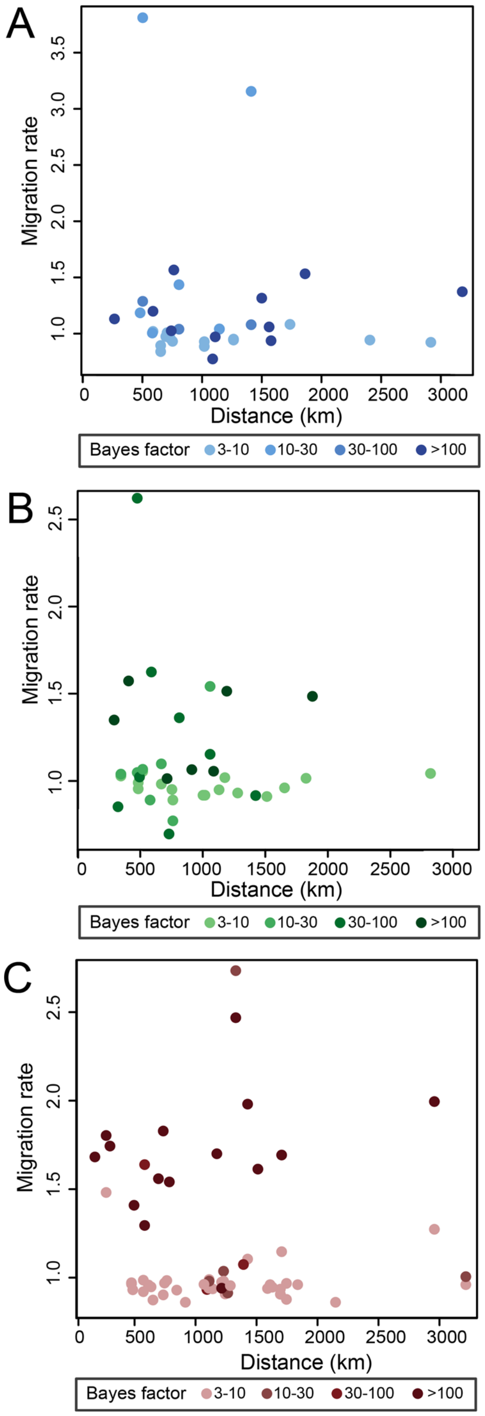

Fig. S13. No associated between lineage migration rates and distances when sequence locations of AIV subtypes H5N1, H5N6 and H7N9 are randomized before phylogeographic analysis using the same method and datasets used in Figure 1. (A) H5N1 (R = -0.052, *P* = 0.765); (B) H5N6 (R = -0.096, *P* = 0.566); and (C) H7N9 (R = -0.096, *P* = 0.566).

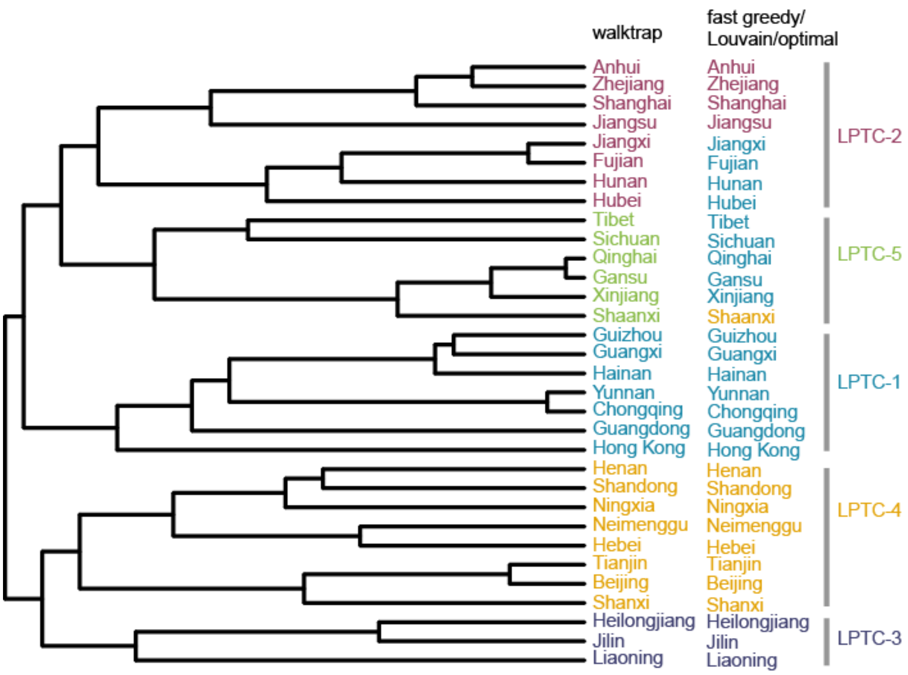

Fig. S14. Community detection using Walktrap, Fast Greedy, Louvain, modularity optimization. The hierarchical structure in the left panel is identified using the Walktrap algorithm. Location are coloured according to the communities detected by Walktrap (left column), and by fast greedy, Louvain and modularity optimization (right column).

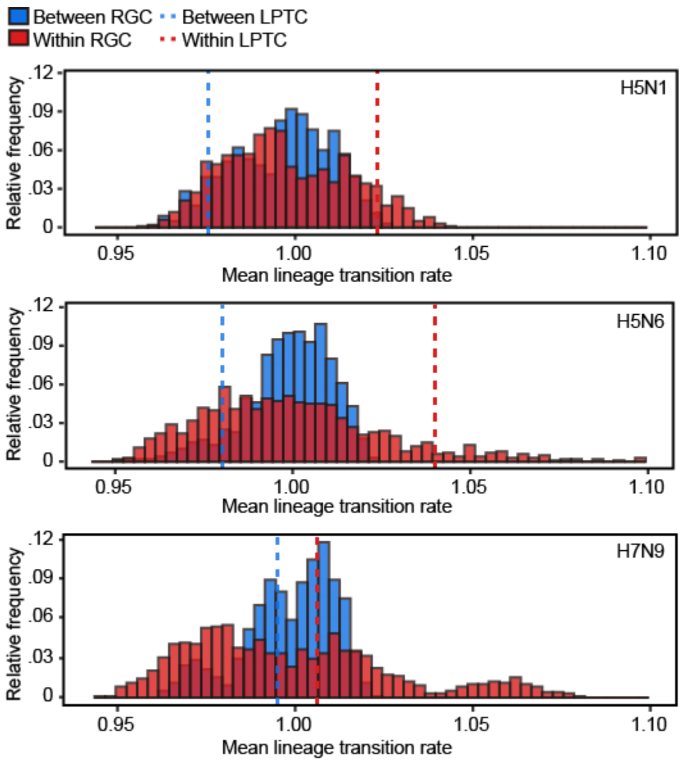

Fig. S15. Histograms of mean AIV lineage transition rates among-location with communities detected by the Fast Greedy, Louvain, modularity optimization. The raw transition rates between locations are obtained from the analyses reported in Fig. 1. Two means are shown here: blue = mean transition rate for pairs of locations in different communities; red = mean transition rate for pairs of locations in the same community. Dotted vertical lines show these two means calculated using the partition of the empirically-derived ("true") LPTC network.

Table S1. Generalized linear model results (controlling for viral sample size)

Predictor	Inclusion*	BF†	cEffect (95% HPD)‡
**H5N1**			
Viral sample size (D)	1	infinite	0.588 (0.370,0.811)
Poultry trade	0.193	9	1.200 (0.678, 1.772)
Bird migration	0.807	149	1.478 (0.925, 2.007)
**H5N6**			
Viral sample size (D)	1	infinite	0.776 (0.442, 1.138)
Poultry trade	0.814	156	0.630 (0.128, 1.194)
Bird migration	0.186	8	0.621 (-0.239, 1.413)
**H7N9**			
Viral sample size (D)	1	infinite	0.871 (0.581, 1.176)
Poultry trade	0.912	367	1.159 (0.315, 2.020)
Bird migration	0.088	3	0.669 (-0.257, 1.477)

*Inclusion, probability that the predictor was included in the model.

†BF, Bayes factor.

‡cEffect, conditional effect size, which represents the estimate of the coefficient conditional on the predictor being included in the model. Both the mean and the 95% highest posterior density credible interval (95% HPD) of the conditional effect size are reported.

Table S2. Out-degree and in-degree of province nodes in live poultry trade network.

	out-degree	in-degree
Beijing	0.0066691	0.0170491
Tianjin	0.0046496	0.0104736
Hebei	0.1070538	0.0637392
Shanxi	0.0163548	0.0382506
Neimenggu	0.007919	0.0149981
Liaoning	0.0389668	0.0246289
Jilin	0.0199215	0.0170071
Heilongjiang	0.0147619	0.0199825
Shanghai	0.0044865	0.0173906
Jiangsu	0.077618	0.0774893
Zhejiang	0.0236046	0.0443643
Anhui	0.0605377	0.067238
Fujian	0.0146695	0.0218523
Jiangxi	0.0398436	0.0369054
Shandong	0.1180784	0.0743098
Henan	0.1252846	0.0848688
Hubei	0.0621741	0.0543692
Hunan	0.0509017	0.0538547
Guangdong	0.0516537	0.0516608
Guangxi	0.0336637	0.0258277
Hainan	0.0055096	0.0042646
Chongqing	0.0171049	0.0215302
Sichuan	0.0530139	0.0454635
Guizhou	0.0111807	0.0244125
Yunnan	0.0136839	0.0222702
Tibet	7.65E-05	0.0007838
Shannxi	0.0101696	0.0319031
Gansu	0.00568	0.0151643
Qinghai	0.0003532	0.0028651
Ningxia	0.0015283	0.0036118
Xinjiang	0.002401	0.00497
Hong_Kong	0.0004851	0.0065007

Table S3. Out-degree and in-degree of province nodes in gene flow network.

	out-degree	in-degree
Hebei	0.046641746	0.046641745
Shanxi	0.013095849	0.013095849
Liaoning	0.046641746	0.046641746
Jilin	0.023406387	0.023235359
Jiangsu	0.034449901	0.033961251
Zhejiang	0.028097438	0.028390628
Anhui	0.050550955	0.050550955
Fujian	0.023504117	0.023650713
Jiangxi	0.040875663	0.040875663
Shandong	0.032959515	0.033350437
Henan	0.072784578	0.072637981
Hubei	0.085074153	0.085171883
Hunan	0.092135161	0.092232891
Guangdong	0.072295928	0.0721249
Guangxi	0.04439395	0.044540547
Chongqing	0.026069535	0.026069535
Sichuan	0.041217719	0.041119988
Guizhou	0.047570183	0.047570182
Yunnan	0.022502382	0.02247795
Shaanxi	0.063133719	0.06313372
Gansu	0.045933201	0.045933201
Xinjiang	0.022942168	0.022991033
Hong_Kong	0.02372401	0.023601847

Table S4. Indicators and data sources of statistics.

Indicator	Mainland China (except HK)	Source	HK	Source
**Poultry population**	RESID[a]	EPS[b]	Maximum Rearing Capacity	ACFD[c]
**Poultry production**	Slaughtered poultry	NBS[d]	Estimated Quantities of Chickens, ducks and quails	ACFD
**Poultry egg production**	Output of poultry eggs	NBS	Estimated Quantities of Hatching Hen Eggs and Table Hen Eggs	ACFD
**Poultry meat production**	Output of poultry meat	EPS	Local production of live poultry	ACFD
**Human population**	Total population	EPS	Total Population	World Bank Open Data
**Poultry egg consumption per capita**	Per capita consumption expenditure of rural households, poultry egg	EPS	Poultry egg consumption / Human population	Calculation of statistics
**Poultry egg consumption**	Poultry egg consumption per capita × Human population	Calculation of statistics	Eggs of wholesale markets throughput	ACFD
**Poultry consumption per capita**	Per capita consumption expenditure of rural households, poultry	EPS	Consumption of live poultry / Human population	Calculation of statistics
**Poultry consumption**	Poultry consumption per capita × Human population	Calculation of statistics	Consumption of live poultry	ACFD

a. RESID is the abbreviation of number of individuals existing at the end of the calendar year(residual poultry);
b. Database: EPS China data (http://www.epschinadata.com/) offering time serial statistical data (including census data) from statistical yearbooks;
c. Agriculture, Fisheries and Conservation Department, The Government of the Hong Kong Special Administrative Region;
d. National Bureau of Statistics;

Additional Dataset S1 (separate file)

Permutation of community structure and comparison**.**

Additional Dataset S2 (separate file)

Accession number of all sequences in this study.

Additional Dataset S3 (separate file)

Original data of predictors for GLM.
